# Tunable Ion-Sensing Using Coulometric-Based Protocols with Permselective Nanomembranes

**DOI:** 10.1021/acs.analchem.5c07283

**Published:** 2026-02-02

**Authors:** Nuria Martínez-Lorca, Yujie Liu, Gregorio Laucirica, Gastón A. Crespo, María Cuartero

**Affiliations:** † 16728 UCAM-SENS, Universidad Católica San Antonio de Murcia, UCAM HiTech, Avda. Andrés Hernández Ros, 1, Murcia 30107, Spain; ‡ Department of Chemistry, School of Engineering Sciences in Chemistry, Biotechnology and Health, 7655 KTH Royal Institute of Technology, Teknikringen 30, Stockholm 100 44, Sweden

## Abstract

Herein, we investigate all-solid-state ion-selective electrodes (ISEs) based on permselective nanomembranes (thickness ∼230 nm) in a coulometric mode. The detection of the potassium ion (K^+^) has been selected as proof of concept, implementing two electrochemical protocols based on the anodic and cathodic readouts of the same ISE. The electrode consists of an ITO glass substrate with the conducting polymer poly­(3-octylthiophene) (POT) electrodeposited on it and a potassium-selective nanomembrane spin-coated over the POT layer. The K^+^ transfer at the membrane-sample interface is mediated by the redox activity of POT, which is in excess with respect to the dopant in the membrane (i.e., the anion part of the cation exchanger, R^–^). In the cathodic protocol, the entry of the K^+^ into the membrane is promoted by the POT^+^ reduction to POT^0^; while in the anodic interrogation, first, K^+^ enters the membrane with a previous accumulation step, and then it is expelled during the oxidation of the POT^0^ to POT^+^. Both protocols were studied under linear sweep voltammetry and chronoamperometry, followed by signal integration to obtain the charge corresponding to K^+^. It is demonstrated that this charge is directly proportional to the K^+^ concentration in the bulk solution. We found two distinct response ranges: 3–20 μM in the cathodic protocol and 200–1000 nM in the anodic one. In addition, the cathodic coulometry strategy revealed excellent repeatability and reversibility within the linear range of response. The developed analytical approach demonstrates suitability in the quantification of real samples, i.e., human urine, horse serum, canal water, and standard KCl solution, while providing a linear and tunable coulometric response over a broad concentration range from the nanomolar to the micromolar level. Moreover, the sensor can be readily integrated into microfluidic devices, additionally offering the advantage of small sample volume requirements. The demonstrated reversibility, along with the ability to customize the ionophore in the membrane for an analysis of different ions, renders the proposed concept adaptable and exceptionally suitable for clinical analysis and environmental monitoring.

## Graphical abstract

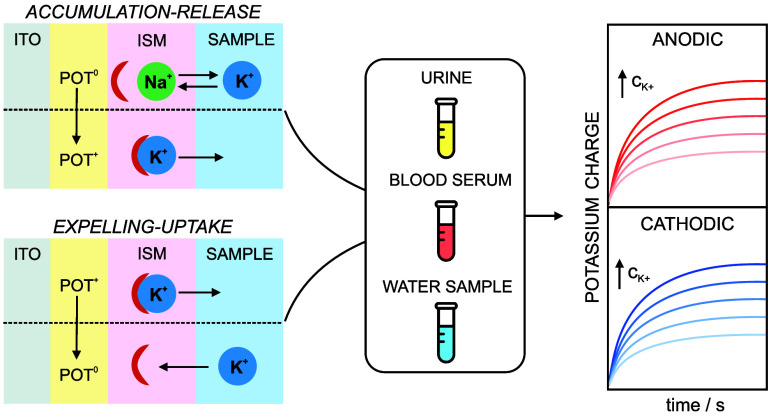

Ion-selective electrodes (ISEs) are a recognized analytical tool for the detection of ions, regardless of their electrochemical activity. While the interrogation at zero current conditions (i.e., traditional potentiometry) has demonstrated significant potential in the digitalization of ion levels in complex matrices,^1–5^ dynamic electrochemical readouts were introduced, aiming to overcome certain analytical limitations. Special emphasis has been dedicated to the improvement of the limit of detection and selectivity. This has been particularly demonstrated for ISEs based on very thin ion-selective membranes (ISMs) backside contacted with the conducting polymer poly­(3-octylthiophene) (POT).^6,7^ In essence, the application of a linear sweep potential imposes a charge delocalization that leads to an ion transfer event (IT) at the sample-ISM interface, which can be exploited for analytical purposes. This mechanism has mainly been proposed for cations (K^+^, Ca^2+^, Li^+^, Na^+^, protamine) and some anions (ClO_4_^–^, hexafluoroarsenate, HCrO_4_^–^).^6–11^

In terms of limit of detection, the membrane can operate under two different regimes, i.e., diffusion-control and thin-layer modes, depending on the concentration of the targeted ion in the solution and how its accumulation in the ISM occurs.^12,13^ The transition between regimes enables the adjustment of the response range of the ISE, hence affecting the limit of detection. Importantly, the exchange capacity of the ISM must be set by the cation-exchanger concentration. Then, when the ISM is immersed in a solution containing the cation analyte, the cation within the exchanger is substituted by the analyte. The extent of this replacement determines the operational regime of the ISE. At low concentrations (nano and micromolar), the replacement is partial and increases with the analyte concentration in the solution according to a diffusion control regime. This translates into a voltammetric peak that increases with the concentration. However, there is a certain analyte concentration at which the replacement is total, which is reflected in a peak shifting to more positive potentials along with the concentration. This behavior is established according to a thin-layer regime.^3^ Notably, fine control of the limit of detection is possible by modulating the accumulation of the analyte from the sample into the membrane and thereby influencing the concentration, thus facilitating the transition between regimes. For example, applying an adequate potential for a relatively long time (ca. 12 min) revealed nanomolar limit of detection for Ag^+^ and K^+^.^12,14^ Restricting the time to seconds, the analysis of micromolar K^+^ concentration was demonstrated, whereas the absence of any accumulation resulted in either micro or millimolar detection range.^12^

Regarding selectivity, it is primarily dictated by the presence of one or more ionophores (i.e., selective receptors) in the ISM. Reports indicate that when the ISM incorporates up to three ionophores, the same ISE can analyze multiple cations. Each of the cations for which the ISM is selective manifests in a peak at a certain potential window (depending on the corresponding cation-ionophore binding constant) that independently evolves with the concentration.^6^ For example, the simultaneous determination of Li^+^, Na^+^ and K^+^ was achieved in undiluted blood using a three-ionophore-based ISM interrogated by cyclic voltammetry.^7^

A key aspect to providing the described features regarding limit of detection and selectivity is the use of a very thin ISM, indeed rather beyond the thin-layer domain (<100 μm in thickness), where charge transfer within this membrane is not restricted by diffusion or mass transport processes.^2^ An influential article by Anson et al. explored charge-transfer mechanisms at a liquid–liquid interface between a sample solution and a thin layer of an organic solvent (nitrobenzene or benzonitrile, approximately 30 μm thick), demonstrating cyclic voltammograms exhibiting thin-layer characteristics for ferrocene.^15^ This preliminary evidence has contributed to ongoing research on voltammetric thin-layer membrane ion-selective electrodes (ISEs).

In subsequent years, Amemiya and coworkers reported on anion extraction with a POT-membrane electrode, the membrane being plasticized PVC of ∼3–4.5 μm thickness.^16^ The oxidation of POT was coupled to the reversible transfer of anions (ClO_4_^–^) from the solution into the membrane. Despite interesting theoretical understanding being provided, irreversible voltammograms were experimentally observed. Si and Bakker proposed an even thinner membrane (340 nm) containing cation and anion exchangers to trigger both cation and anion transfers, i.e., Na^+^ and Cl^–^.^17^ While the cation transfer was totally reversible, the anion one found certain irreversibility. In all these cases, potential sweep techniques were applied to investigate changes in the peak current or position for analytical purposes. More recently, the voltammetric signals from the cathodic and anodic peaks were evaluated charge-wise (i.e., coulometry readout) when further coupled to thin-layer samples.^18,19^

Beyond cyclic voltammetry, and considering or not a coulometry interpretation, chronoamperometry and chronopotentiometry have been proposed for analogous ISE systems on a few occasions. Bobacka and coworkers, as well as Wei and coauthors, have been the groups who have worked the most in these conditions, as far as we know. Constant potential coulometric readout has been proposed relying on the capacitance of the solid contact element (e.g., polyaniline or PEDOT) to detect both anions (Cl^–^, NO_3_^–^, SO_4_^2–^, ClO_4_^–^) and cations (Ca^2+^, Pb^2+^, K^+^, H^+^).^20–23^ Some studies showed that increasing the thickness of the conducting polymer while reducing the membrane thickness (e.g., from 60 μm to 200 nm), resulted in the amplification of the cumulated charge.^24^ This was found to enhance in turn the sensitivity and detection limit of the corresponding ISE via modulation of the Nernstian slope along with the capacitance of the solid-contact.

Chronopotentiometric approaches involving the application of current pulses for biosensing^25^ and seawater analysis^26^ have also been proposed. The strategies not only significantly reduced the recovery time compared to traditional zero-current methods but also, it was found to enhance sensitivity and selectivity. The Cu^2+^ trace analysis in the presence of a high electrolyte background was achieved with a solid-contact ISE comprising a ∼4 μm-thick membrane free of any ion exchanger.^27^ Furthermore, potential-based approaches for pH and nitrate sensing were considered, enabling the precise measurement of small concentration changes.^28,29^ All these experimental results have shown that chrono-based methods can provide improved analytical performance (i.e., amplified analytical signal, better signal-to-noise ratio and shorter response time) compared to traditional potentiometric ISEs.

In this work, we present a coulometric methodology for K^+^ detection, as proof of concept, in tunable concentration ranges using two protocols based on the anodic and cathodic interrogation of POT-membrane electrodes. K^+^ combines biological relevance, experimental robustness, and well-established electroanalytical chemistry, making it an ideal model ion for demonstrating the fundamental principles, absolute accuracy and feasibility of a new coulometric ion-selective sensor. Truly, since the thermodynamic and kinetic properties of ISEs for K^+^ are well-known, it is easier to attribute the observed effects to the new coulometry methodology rather than to the membrane chemistry. The anodic protocol involves an initial accumulation step to force K^+^ entering the membrane, followed by its expulsion to the sample during the controlled oxidation of POT. Conversely, the cathodic method initially depletes the membrane of any cation present in it and then, K^+^ is directly transferred from the sample into the membrane. Specifically, the developed K^+^-selective electrode is based on a nanomembrane interrogated via linear sweep voltammetry (LSV) and chronoamperometry, with subsequent signal integration to quantify the charge. After a thoroughly analytical evaluation of both techniques, chronoamperometry reveals a better agreement between the measured charge and the analyte concentration compared to LSV. To the best of our knowledge, the present study represents the first combination of potentiostatic coulometry with all-solid state ISEs based on a conducting polymer in contact with a nanometer-sized membrane. Importantly, the comparative study of anodic and cathodic protocols provides insights into energetic and kinetics differences of ion-transfer processes occurring in both directions across the sample-membrane interface. The suitability of the developed sensor for real sample measurements is demonstrated on various biofluids and environmental waters. Notably, the established coulometric approach can be adapted to other ions by optimizing the membrane composition.

## Experimental Section

### Reagents, Materials, and Equipment

Lithium perchlorate (anhydrous, 99%, LiClO_4_), acetonitrile (anhydrous, ≥ 99.5%, ACN), absolute ethanol (≥99.5%), potassium chloride (99.5%) and potassium chloride standard solution (0.1 M) were purchased from VWR. 3-octylthiophene (97%, OT), tetrahydrofuran (anhydrous, ≥ 99.9%, THF), polyurethane (PU, Selectophore), bis­(2-ethylhexyl)­sebacate (DOS), sodium tetrakis­[3,5-bis­(trifluoromethyl)­phenyl]­borate (NaTFPB), potassium ionophore I (Valinomycin) and high-purity grade sodium chloride Selectophore were sourced from Sigma-Aldrich. All reagents were used as received. Aqueous solutions were prepared by dissolving the appropriate salts in deionized water (>18.2 MΩ·cm). Indium tin oxide (ITO) coated glass slides (25 mm × 25 mm × 1.1 mm, transmittance >83%, surface resistivity <10 Ω/sq) were acquired in Zhuhai Kaivo Optoelectronic Technology.

Linear sweep voltammetry and chronoamperometry experiments were performed with a VIONIC potentiostat controlled with INTELLO 1.5 software (Metrohm). The 801-stirrer acquired from Metrohm was used to stir the solution. A 940 Professional ion chromatography (IC) instrument equipped with a Metrosep C6-150/4.0 column and 889 IC Sample Center as Autosampler (injection volume of 20 μL, sample eluent of 4 mM HNO_3_, flow rate of 0.9 mL/min) was employed to validate the proposed methodology with real samples. Calculations were accomplished using MATLAB_R2022b software.

The K^+^ content of various samples was analyzed: blood serum, urine, canal water and standard solution. All samples were adequately refrigerated before being analyzed and filtered using 0.45 μm pore size filters connected to syringes to eliminate larger particles, except for the standard KCl solution. The standard 0.1 M KCl solution was acquired in VWR (reference 87898.290). The horse serum sample was purchased from Sigma-Aldrich (reference H1270-100 ML). The urine sample was obtained from healthy volunteers (Ethical permit CE062308). The canal water sample was collected in “El Albujón” (37°42′58”N 0°51′38”W, Region of Murcia, Spain). For the electrochemical measurements, all samples were diluted in 10 mM NaCl electrolyte at varying proportions to guarantee adequate conductivity for the more diluted samples and to remain within the linear response range established by the developed method. The samples were also analyzed with IC. The serum sample was additionally deproteinized by mixing four parts of absolute ethanol with one part of serum. This mixture was then centrifuged in a microcentrifuge model MicroStar 12 at 6000 rpm for 10 min.

### Preparation of the K^+^-Selective Electrode

The ISE for K^+^ was based on an ITO-POT-membrane configuration. The ITO glass was cleaned in ethanol using ultrasonicator (USC200TH, VWR) for 10 min, being then dried with a mild nitrogen flow. The ITO was first modified with a POT film. For that, the ITO was positioned in a custom-made electrochemical cell reported elsewhere,^30^ with a Pt rod (2 mm in diameter) as the counter electrode and Ag/AgCl wire as pseudoreference electrode, and a 0.1 M solution of LiClO_4_ and 3-octylthiophene in ACN was added. A nitrogen flow was passed for 15 min to degas the solution. Then, the POT film was formed via electropolymerization on the ITO surface (cyclic voltammetry from 0 to 1.5 V, scan rate of 100 mV s^–1^, 2 scans). Finally, the POT film was discharged at 0 V for 120 s. The obtained film was immersed first in ACN and later in THF for 30 min and 10 s, respectively. The ITO-POT electrode was dried with a soft nitrogen flow. Subsequently, the membrane cocktail was prepared by dissolving 20 mg of PU, 20 mg of DOS, 0.8 mg of NaTFPB and 2 mg of potassium ionophore I in 2 mL of THF. A volume of 30 μL of this cocktail was deposited on the ITO-POT electrode by spin coating (1500 rpm, 120 s) with a spin coater model WS-650-23B acquired from Laurell Technologies. With this procedure, a nanomembrane of ca. 230 nm in thickness (calculated by ellipsometry) was obtained.^31^ This ultrathin configuration eliminates the need for a prior conditioning step, since ion equilibration occurs upon immersion of the nanomembrane in the sample solution, in a matter of seconds.^32^ Parameters related to electrode architecture, including the membrane composition (e.g., ion-exchanger content,^7^ overall composition^31^) as well as capacitance and thickness of the solid contact element^33^ were investigated and optimized in previous works. The absence of water layer formation was extensively studied in a previous work.^31^

### Protocols for the Electrochemical Measurements

The electrochemical cell used for the measurements was reported elsewhere.^12^ Briefly, the cell consists of two metallic covers designed to hold two ITOs, with one serving as the working electrode and the other functioning as a window to check for the absence of bubbles in the system. The central part of the cell is an acrylic compartment, sealed with O-rings to prevent leaks. Inside this compartment, the pseudoreference (Ag/AgCl wire) and the counter (Pt rod) electrodes are immersed in a volume of 8 mL of background electrolyte. The influence of different background electrolytes on the K^+^ response was previously investigated, revealing no significant differences in the position or magnitude of the voltammetric peak, with a clear separation between the K^+^ peak and those associated with background cations.^7^ Notably, this behavior facilitates the coulometric interrogations herein explored, since the potential pulses can be precisely applied for each ion-transfer event. A scheme of the electrochemical cell together with a real picture of the experimental setup are shown in Figure S1.

The protocol for the anodic coulometry interrogation of the ITO-POT-membrane electrode is termed as A-IT-C (Figure 1). This approach involved an initial accumulation step at −0.2 V for 450 s while the sample was stirred at 100 rpm. Subsequently, an anodic linear sweep from −0.2 to 1.1 V at a scan rate of 50 mV/s was applied. The related voltammogram reveals certain peaks in connection to generated ion transfers at the membrane-sample interface, allowing to identify and select the potential needed to expel each cation in a further anodic chronoamperometry interrogation. In the case of K^+^ measurements in NaCl background, three different potentials are applied in pulses of 40 s after the accumulation step at −0.2 V for 450 s. First, a potential at which there is no ion transfer is applied to account for a baseline correction (*E*_baseline_). Second, a potential to expel any background cation, in this case Na^+^ from the membrane, is applied (*E*_Na_). Third, a potential to release the K^+^ from the membrane to the solution is applied (*E*_K_). Finally, the dynamic current signal associated with each potential pulse is integrated to obtain the charge associated with the Na^+^ and K^+^ transfers. The described A-IT-C protocol is illustrated in Figure 1 together with the signal treatment (further details about the charge calculation from the voltammograms and chronoamperograms are available in the Supporting Information, Figure S2 and Figure S3.

**Figure 1:**
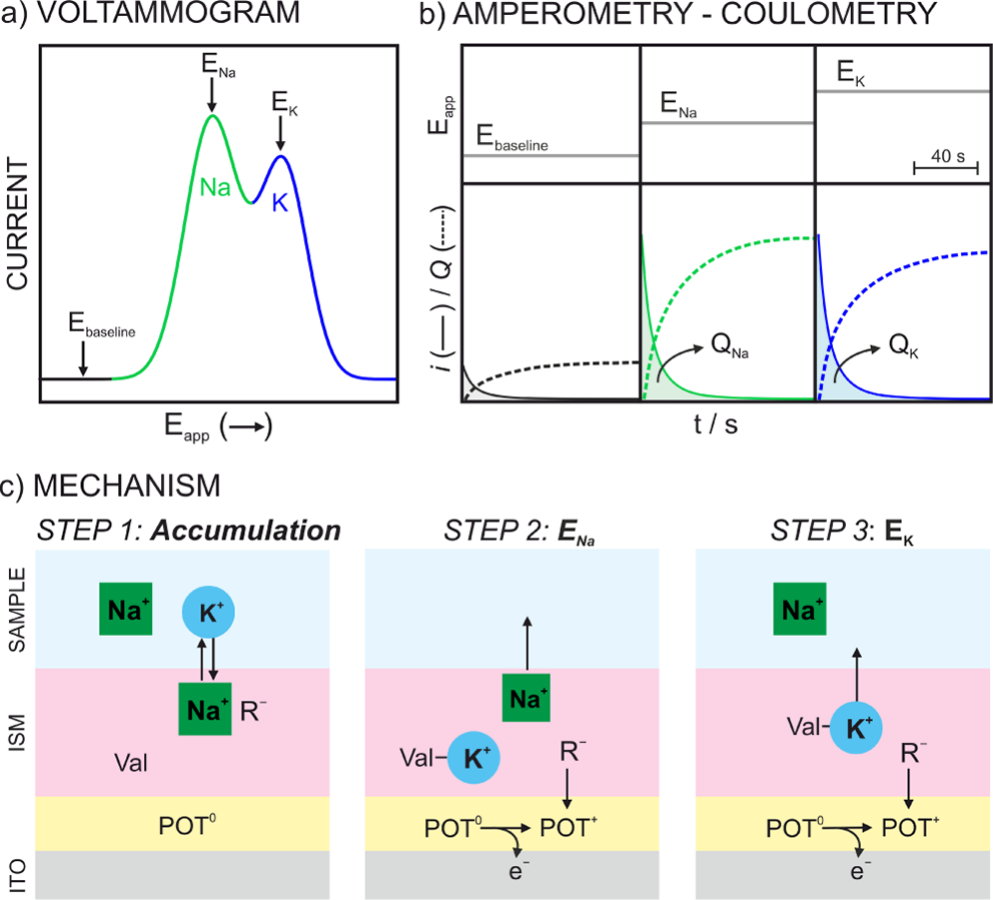
Schematic illustration of the A-IT-C protocol in the ITO-POT-membrane electrode. (a) Anodic voltammogram for a solution containing Na^+^ and K^+^. (b) Amperograms (solid line) and cumulated charge (dash line) obtained when voltages equivalent to peak potentials in the voltammogram are applied. (c) Working mechanism at each applied potential. ISM = ion-selective membrane. R^–^ = TFPB^–^. Val = valinomycin.

The protocol for the cathodic coulometry interrogation of the ITO-POT-membrane electrode is named as C-IT-C (Figure 2). This approach involved an initial depletion of any cation present in the membrane. The step consisted of applying 1 V for 150 s while the sample was stirred at 100 rpm. Subsequently, a cathodic linear sweep from 1.1 to −0.1 V at a scan rate of 5 mV s^–1^ was applied. Note that a scan rate lower than that used in the anodic interrogation was needed because more time was required for the related transfers providing well-differentiated peaks in the voltammogram. Thus, the voltammogram should permit to identify and select the potential needed to uptake the K^+^ from the sample into the membrane in a further cathodic chronoamperometry interrogation. In the case of K^+^ measurements in NaCl background, two different potentials are applied in pulses of 40 s. First a potential at which there is no ion transfer is applied to account for a baseline correction (*E*_baseline_). Second, a potential to directly uptake the K^+^ present in the solution is applied (*E*_K_). Remarkably, K^+^ can be directly accumulated into the membrane without the need to accumulate Na^+^. Finally, the dynamic current signal associated with each potential pulse is integrated in absolute value to get the charge associated with the K^+^ transfer.

**Figure 2:**
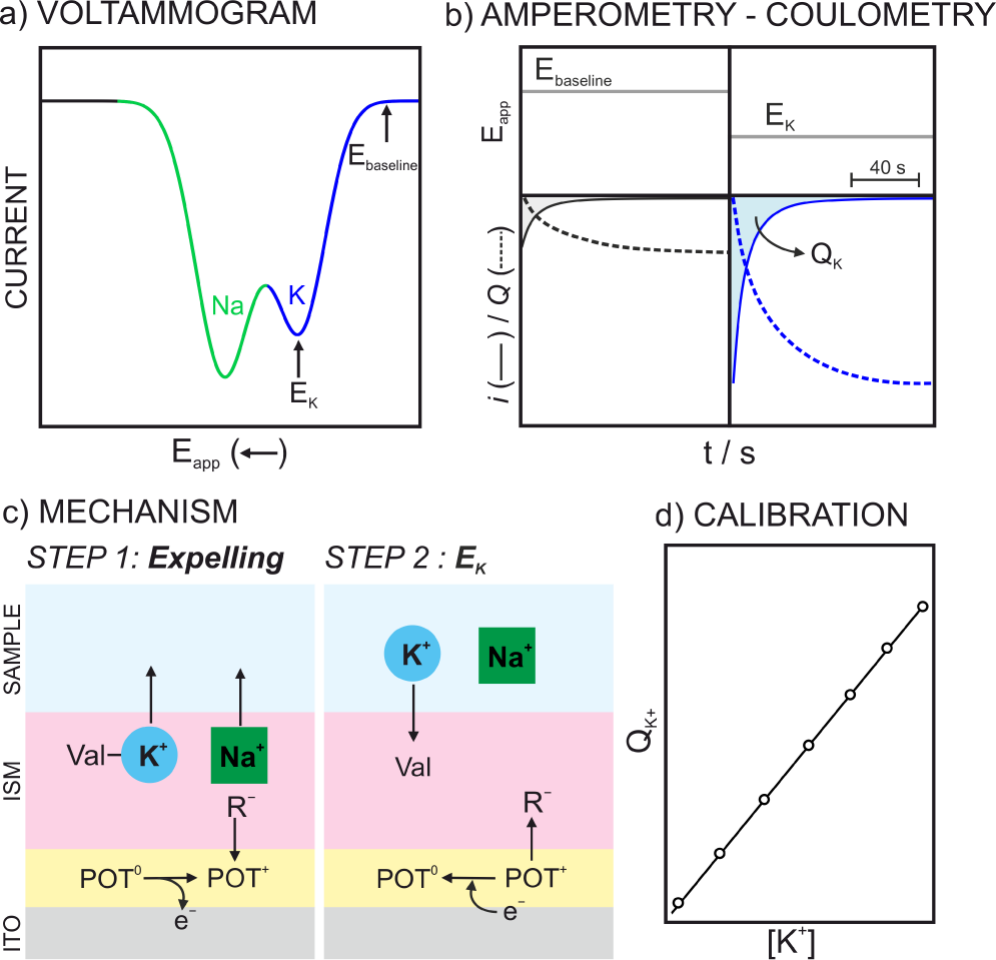
Schematic illustration of the C-IT-C protocol in the ITO-POT-membrane electrode. (a) Cathodic voltammogram for a solution containing Na^+^ and K^+^. (b) Amperograms (solid line) and cumulated charge (dash line) obtained when voltage equivalent to peak potential in the voltammogram are applied. (c) Working mechanism at each applied potential. (d) Expected linearity of the K^+^ charge with the concentration. ISM = ion-selective membrane. R^–^ = TFPB^–^. Val = valinomycin.

## Results and Discussion

### The Mechanism and Its Premises

This work investigates two different protocols (A-IT-C and C-IT-C) to generate selective IT events at the interface of a nanomembrane with a sample solution. The nanomembrane was added on top of an ITO-POT electrode that allows imposing a charge delocalization that ultimately drives the IT. The two methods involve a sequence of interconnected charge transfer processes across the different interfaces conforming the ITO-POT-ISM system.

The cation initially present in the membrane (without facing any sample solution) is Na^+^, which comes from the cation-exchanger (Na^+^TFPB^–^). When the membrane is immersed in a sample solution containing K^+^, the immediate exchange of Na^+^ by K^+^ occurs because of the high selectivity of the membrane toward K^+^ and the despair concentration gradient. Thus, there will be a Na^+^/K^+^ replacement degree depending on the K^+^ concentration in the solution and the duration required to establish equilibrium at the sample/membrane interface. Correspondingly, considering the following situations that (i) only Na^+^ is present in the membrane (no K^+^ in the sample solution), (ii) both Na^+^ and K^+^ are present (increasing amount of K^+^ in the sample solution), or (iii) only K^+^ (at a relatively high K^+^ concentration, close to 1500 nM), the resultant IT voltammograms will exhibit one, two or one peak respectively, with the peak corresponding to Na^+^ occurring at a lower potential than that of K^+^. This is because K^+^ is stronger retained in the membrane than Na^+^ due to the ionophore.^7^ A concentration situation displaying both peaks is adequate evidence to select those potentials propitiating either Na^+^ or K^+^ transfer (*E*_Na_ and *E*_K_) to build up a constant-potential coulometry detection method for K^+^, as herein proposed. The transfer potentials can be indiscretely selected considering increasing K^+^ concentration in the sample solution, because the peak potential did not significantly change (ca. ±50 mV) and sufficient time is provided for the described shift equilibrium (40 s). Both anodic and cathodic procedures can, in principle, be employed, which are thoroughly examined as follows.

For the A-IT-C, it is convenient to ensure the POT film is mainly in the basal state POT^0^ to maximize the described replacement. For such a purpose, a sufficiently negative potential is first applied in the electrochemical protocol: this indirectly drives an accumulation of K^+^ in the membrane upon time contact with the sample (Step 1 in Figure 1c). Then, when *E*_Na_ is applied, the POT^0^ film is oxidized to POT^+^; this latter is stabilized with the TFPB^–^ present in the membrane, and finally, to maintain the electroneutrality, Na^+^ ions present in the membrane are expelled to the solution (Step 2 in Figure 1c). Once Na^+^ has been expelled, K^+^ can be subsequently released by the application of a higher potential *E*_K_ (Step 3 in Figure 1c). In contrast, the C-IT-C protocol starts with a polarization at 1 V, which leads to the POT film oxidation to POT^+^ and the expelling of all the cations present in the membrane to the solution (Step 1 in Figure 2c). The following step consists of applying *E*_K_ to control the flux of K^+^ from the solution to the membrane, with TFPB^–^ returning to the membrane to maintain the electroneutrality (Step 2 in Figure 2c).

It is here anticipated that tailoring the A-IT-C and C-IT-C protocols results in two different response ranges when analyzing the coulometry output from steps iii and ii respectively: nanomolar and micromolar concentrations of K^+^. The current responses are integrated to acquire the charge and ultimately relate this to the concentration (Figure 2b). Then, the differences expected in the linear response ranges are attributed to the distinct mechanisms involved: The A-IT-C protocol consists of the prior K^+^ accumulation in the membrane followed by its release, whereas the C-IT-C protocol relies on the initial K^+^ expulsion followed by its entry. These processes inherently differ in energy requirements and thus K^+^ transfer efficiency: the K^+^ uptake into the membrane is generally more favorable than its release due to the presence of the ionophore. Additionally, the accumulation step in A-IT-C protocol allows for amplifying the signal, particularly at low concentrations. Therefore, we expect the anodic protocol to enhance sensitivity, while the cathodic method exhibits a wider linear response.

The developed coulometric approach provides a readout under equilibrium conditions, meaning that the target ion is not totally depleted from the sample and hence, the measured charge reflects a fraction of the analyte present in the sample. The response is independent of the equilibrium potential at the membrane-sample interface, which avoids the traditional logarithmic dependence on ion activity and minimizes (even suppresses) the influence of temperature or ionic strength variations. These characteristics enhance sensitivity and long-term stability while reducing the need for recalibration compared to traditional potentiometric ISEs. Furthermore, the method must in principle exhibit lower sensitivity to the reference electrode. Regarding selectivity, it is anticipated that the application of a potential exclusively related to K^+^ transfer helps suppressing potential interferents (e.g., NH_4_^+^) when considering the potentiometric selectivity coefficients.

### Investigation of the A-IT-C Protocol

The A-IT-C protocol was studied by increasing the K^+^ concentration in the range of 50–3000 nM in 10 mM NaCl background solution. First, the electrode was interrogated under LSV in the potential window from −0.2 to 1.1 V. The baseline-corrected voltammograms are presented in Figure 3a (raw data are available in Figure S4). A peak centered at 511.1 mV, attributed to Na^+^, was observed for the background solution and showed a progressive decreased upon K^+^ additions. This was accompanied by the appearance of a second peak at 759 mV associated with K^+^, which increased with the increament of K^+^ concentration in the sample solution. These trends can be observed in Figure 3b. Notably, a small peak at 764 mV was already present in the initial background measurement, indicating minor K^+^ contamination in the starting solution (∼100 nM). This residual signal was considered and systematically subtracted in the subsequent analyses. Additionally, the Na^+^ peak exhibited a shoulder in the range of 305–390 mV, which may be attributed to the existence of two distinct spatial conformations of the POT. Then, when a concentration of 3000 nM was present in the sample, only the K^+^ peak was observed, which suggested the membrane saturation with K^+^.^31^ In essence, all the positive charge positions available in the membrane that initially were occupied by Na^+^ (charge of 20.9 μC) were substituted by K^+^ (charge of 20.8 μC).

**Figure 3:**
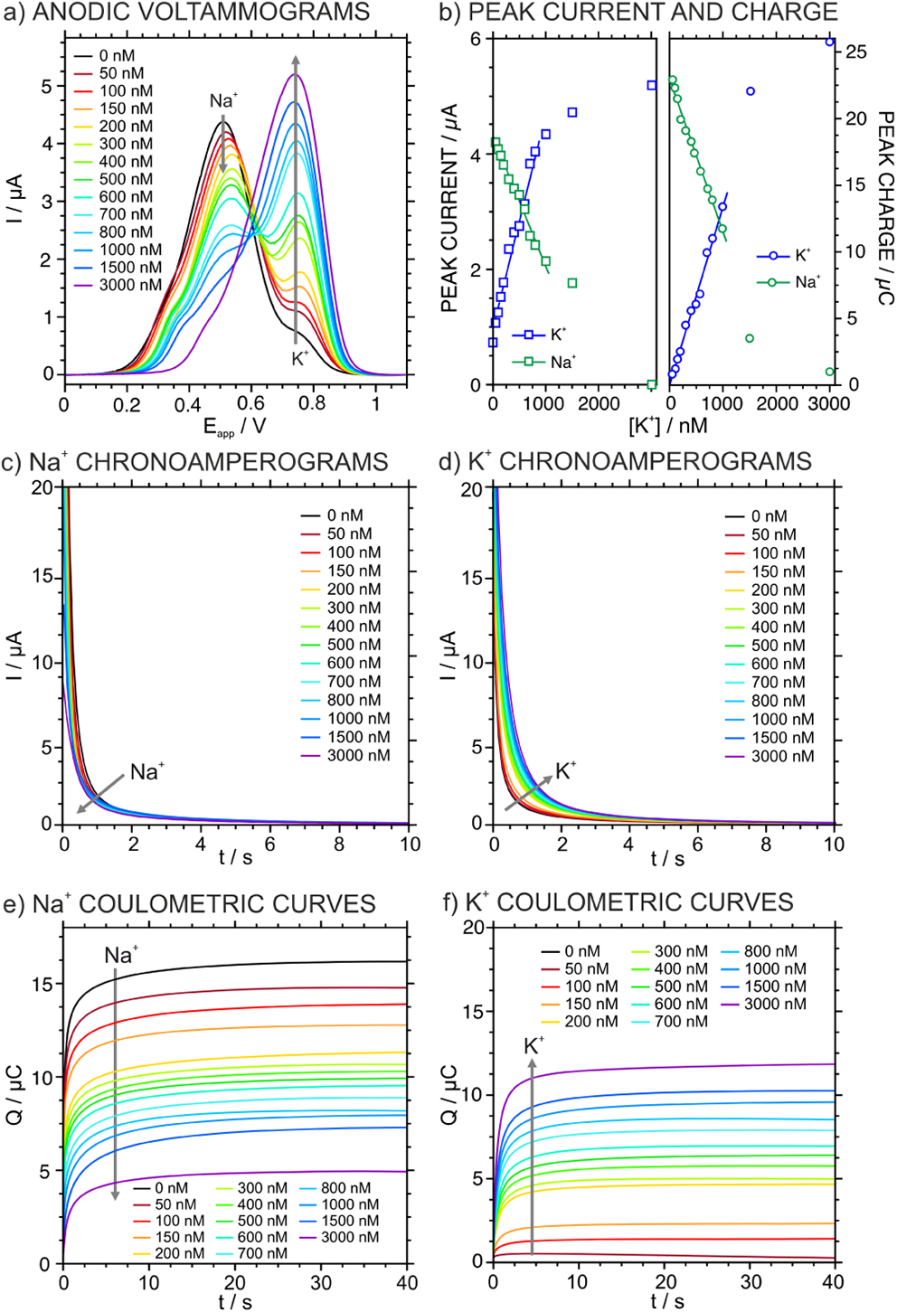
(a) Anodic LSVs at increasing KCl concentrations in 10 mM NaCl, scan rate = 50 mV s^–1^ (b) Plot of the peak currents and peak charges for Na^+^ and K^+^ versus the K^+^ concentration. (c) Chronoamperograms for Na^+^ at increasing KCl concentrations in 10 mM NaCl. (d) Chronoamperograms for K^+^ at increasing KCl concentrations in 10 mM NaCl. (e) Coulometric corrected curves for Na^+^ at increasing KCl concentrations in 10 mM NaCl. (f) Coulometric corrected curves for K^+^ at increasing KCl concentrations in 10 mM NaCl.

A linearity was observed for the peak current and charge within certain ranges of K^+^ concentration: *I*_K_^+^ (μA) = 4 × 10^–3^ *c*_K_^+^ (nM) + 0.8916, *R* ^2^ = 0.9845 (LRR: 50–800 nM) and *Q*_K_^+^ (μC) = 1.34 × 10^–2^ *c*_K_^+^ (nM) – 7.4 × 10^–3^, R^2^ = 0.9870 (LRR: 50–1000 nM). These results indicate that both, the peak current and charge could be used from an analytical point of view with the charge covering a wider K^+^ concentration. Beyond this, the LSV results (Figure 3a,b) were indicative for selecting the *E*_baseline_, *E*_Na_ and *E*_K_ to be applied in the chronoamperometry readout. These potentials were chosen to be 100, 500, and 750 mV respectively. Small variations in these values (5% for the Na^+^ and 2% for the K^+^ peak) were not significant in the chronoamperometry interrogation, as sufficient time was allowed for the current to stabilize.

Following the LSV part, the accumulation step was repeated and *E*_baseline_, *E*_Na_ and *E*_K_ were subsequently applied for pulses of 40 s each. The corresponding chronoamperograms are presented in Figure 3c, Figure 3d, and Figure S5. The integration of the latter two current signals over time yielded the charge associated with each ion transfer process. Then, the charge attributed to nonfaradaic processes (primarily capacitive currents, typically within the range of 1.0–1.5 μC), was estimated from the application of *E*_baseline_. This value was subtracted from the signals corresponding to Na^+^ and K^+^ transfer steps, allowing for the determination of the net ion transfer charges. Consistent with the data analysis performed for LSV measurements, the residual K^+^ signal observed in the background, likely due to minor contamination, was also subtracted from all coulometric curves of K^+^.

Then, the integration of the current decays related to Na^+^ and K^+^ derives in the corresponding charge–time curves presented in Figure 3e and Figure 3f. An increase in the total charge for K^+^ with a simultaneous decrease of that for Na^+^ was found as the K^+^ concentration was increased in the sample solution. Moreover, this corresponded well with the total K^+^ charges calculated in the previous LSV experiment, obtaining an acceptable correlation when considering all data points (Pearson coefficient of 0.904), and being even better when the three lowest concentration points were excluded (Pearson coefficient of 0.958), as shown in Figure S6a. The deviations at lower charges (and hence lower concentrations) can be attributed to higher errors associated with the peak charge calculation. In any case, the maximum difference between the two techniques within the LRR was 4 μC.

A linear correlation was found in the concentration range from 200 to 1000 nM: *Q*_K_^+^ (μC) = 6.50 × 10^–3^ *c*_K_^+^ (nM) + 3.206, *R* ^2^ = 0.9938 (see below, Figure 5a). Compared to the LSV data (Figure 3b), where the charge exhibited a linear range of 50–1000 nM and the peak current of 50–800 nM, the charge from chronoamperometry showed slightly superior linearity and excellent consistency across the higher concentrations that were tested. Although the sensitivity of the LSV-charge method (slope = 1.34 × 10^–2^ μC/nM) was greater than that of the amperometry-charge method (slope = 6.50 × 10^–3^ μC/nM), the chronoamperometry allows for simpler data processing. Specifically, it eliminates the need for peak deconvolution and is less influenced by peak shifts or waveform characteristics, enhancing its reproducibility.

### Investigation of the C-IT-C Protocol

The C-IT-C protocol was studied by increasing the K^+^ concentration in the range of 1–50 μM in 10 mM NaCl background. The electrode was initially interrogated with LSV within a potential window from 1.1 to −0.1 V. The baseline-corrected voltammograms are presented in Figure 4a, being the uncorrected and original data available in Figure S7 in the Supporting Information. A single peak at 521.9 mV was observed in the absence of K^+^, corresponding to Na^+^. Upon increasing K^+^ concentrations, the Na^+^ peak progressively decreased, while a second peak appeared at 0.73 V, associated with the K^+^ entry into the membrane. These trends are reflected in Figure 4b for the peak currents and related charges for K^+^ (the analysis of the peak current was not accurate enough in the case of Na^+^ because of a high degree of overlapping with the K^+^ peak). Then, when a concentration of 20 μM was added, only the K^+^ peak was observed. Essentially, the positive vacancies in the membrane, which were initially occupied by Na^+^ (charge of 21.7 μC) are entirely replaced by K^+^ (charge of 23.9 μC) from that concentration.

**Figure 4:**
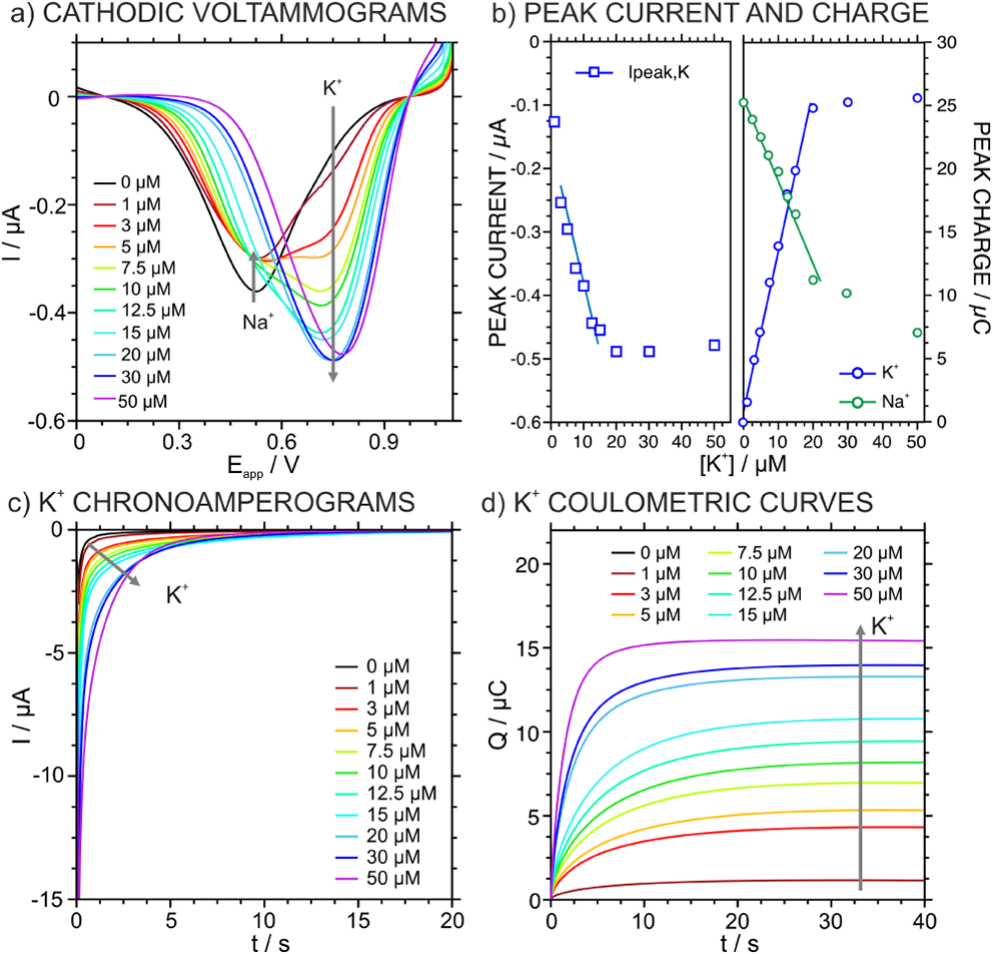
(a) Cathodic baseline-corrected LSVs at increasing KCl concentrations in 10 mM NaCl, scan rate = 5 mV s^–1^ (b) Plot of the peak currents and peak charges for K^+^ versus the K^+^ concentration. (c) Chronoamperograms for K^+^ at increasing KCl concentrations in 10 mM NaCl. (d) Coulometric corrected responses for K^+^ at increasing KCl concentrations in 10 mM NaCl.

A linear relationship was observed for both the peak current and charge with the K^+^ concentration: *I*_K_^+^ (μA) = −1.74 × 10^–2^ *c*_K_^+^ (μM) – 0.211, *R* ^2^ = 0.9726 (LRR: 3–15 μM) and *Q*_K_^+^ (μC) = 1.330 *c*_K_^+^ (μM) + 0.6459, *R* ^2^ = 0.9947 (LRR: 1–15 μM). A comparison of the linear ranges reveals that both analytical signals exhibited similar results. However, in contrast to the A-IT-C protocol, the peak current seems to be the better choice considering the sensitivity. This can be attributed to the protocol followed for the charge calculation, since in the backward scan the peaks are slightly worse defined than in the forward scan, which reduces the accuracy of the Gaussian fitting. Furthermore, the LSV results were used to set *E*_baseline_ and *E*_K_ to be applied in the chronoamperometry interrogation: 950 and 750 mV, respectively. The minor variations observed in these potentials were considered negligible to affect the chronoamperometric readout providing sufficient time to ensure effective ion transfer (3% for the K^+^ peak with increasing concentrations).

Following the initial depletion step, *E*_baseline_ and *E*_K_ were sequentially applied for 40 s each, and the resulting chronoamperograms are shown in Figure S8 and Figure 4c. Figure 4d presents the corresponding coulometric curves related to K^+^, revealing an increment as the K^+^ concentration increased in the sample solution. As in the A-IT-C protocol, the charge coming from nonfaradaic processes, as estimated from the application of *E*_baseline_, was subtracted from the total charge associated with the K^+^ transfer. Furthermore, the K^+^ signal derived from the background was subtracted from all curves to ensure each measurement started from a baseline corresponding to a fully corrected signal. This corresponded well with the total charges calculated in the previous LSV experiment, obtaining an acceptable correlation when considering all data points (Pearson coefficient of 0.9903), and an even better correlation when four concentration points were excluded (Pearson coefficient of 0.9966), as shown in Figure S6b.

Figure 5b depicts the total charge for K^+^ obtained from the experiment shown in Figure 4d with the K^+^ concentration. A strong linearity was found over the range of 3–20 μM, described by the equation: *Q*_K_^+^ (μC) = 0.5285 *c*_K_^+^ (μM) + 2.813, *R* ^2^ = 0.9988. In comparison with the LSV data, the chronoamperometric method displayed a higher correlation coefficient and a broader linear range for charge detection. This can be attributed to the nature of the charge calculation in chronoamperometry, which is directly obtained by integrating the current response in terms of time. In contrast, calculating the charge from LSV requires complex processing involving Gaussian deconvolution of the overlapping peaks, which may introduce errors in the final charge calculation.

**Figure 5:**
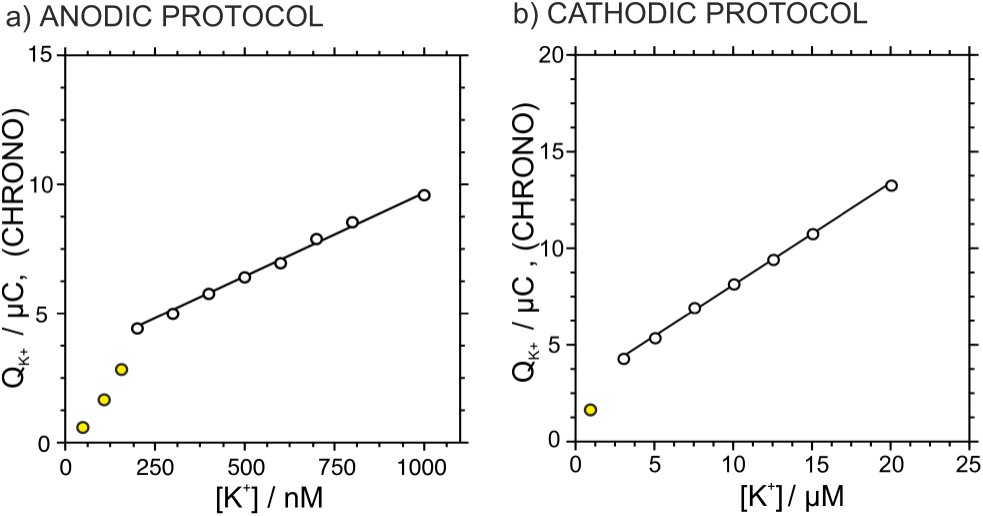
Calibration curves for (a) anodic and (b) cathodic protocols obtained from Figures 3f and 4d, respectively.

### Repeatability and Reversibility Features of the A-IT-C and C-IT-C Protocols

To evaluate the repeatability of the system, three repeated measurements were conducted using the same ITO-POT-membrane electrode. Two K^+^ concentrations within the corresponding LRR were tested for each protocol: 50 and 1000 nM for the A-IT-C protocol and 1 and 12.5 μM for the C-IT-C. In this analysis only K^+^ response was evaluated, since it is the one later considered for analytical exploitation. As shown in Figure 6, the K^+^ amperometric and coulometric responses exhibited a significant overlap regardless of the used protocol. Particularly, minor variations were identified (Table S1 in the Supporting Information). The standard deviations for charges calculated for both concentrations studied with each protocol were consistently low. Thus, the relative standard deviations (RSD) in the determination of the charge remained <1.5%. No significant differences in repeatability were observed between the two methods, confirming that both protocols exhibit reliable and reproducible performance.

**Figure 6:**
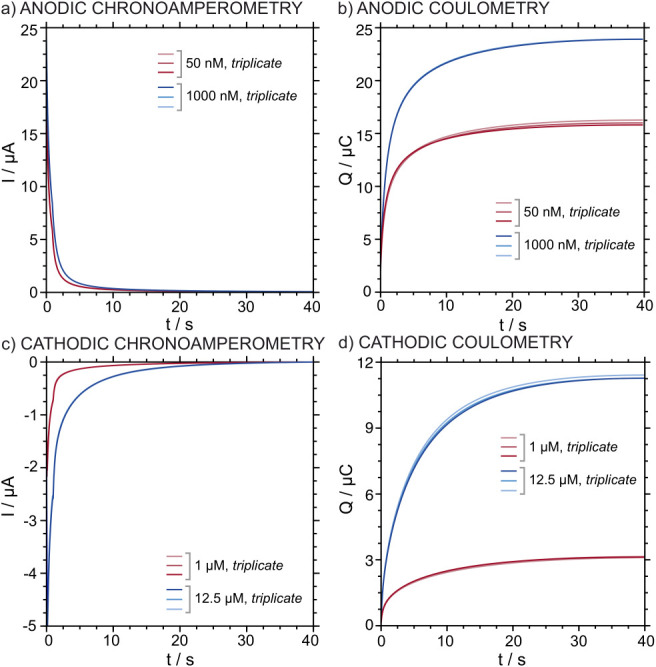
(a) Anodic chronoamperograms obtained in triplicate for two K^+^ concentrations, 50 and 1000 nM in 10 mM NaCl using the same ITO-POT-membrane electrode. (b) The corresponding anodic coulometric corrected curves. (c) Cathodic chronoamperograms obtained in triplicate for two K^+^ concentrations, 1 and 12.5 μM in 10 mM NaCl using the same ITO-POT-membrane electrode. (d) The corresponding cathodic coulometric corrected curves.

The reversibility of the chronoamperometric response was studied using the same ITO-POT-membrane electrode by alternately testing two very different concentrations of K^+^: 50 and 1000 nM for A-IT-C, 1 and 10 μM for C-IT-C. The chronoamperometric and corresponding coulometric responses are presented in Figure 7. Both protocols presented acceptable reversibility, as reflected by the RSD calculated for the charge: ca. 10% (Table S2, Supporting Information). Consistent with the repeatability study, no statistically significant differences were observed between both protocols.

**Figure 7:**
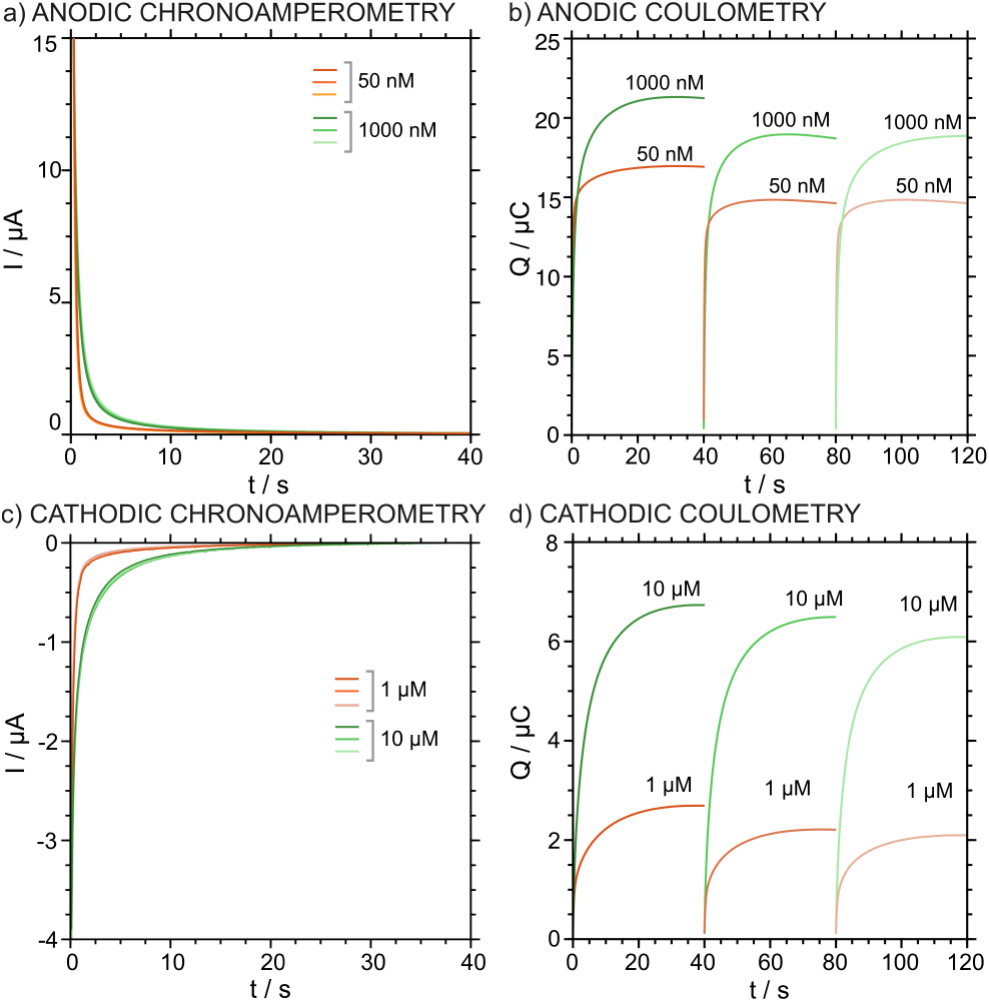
(a) Anodic chronoamperograms obtained by alternating two K^+^ concentrations, 50 and 1000 nM in 10 mM NaCl using the same ITO-POT-membrane electrode. The following sequence was followed: 50 → 1000 → 50 → 1000 → 50 → 1000 nM KCl. (b) The corresponding anodic coulometric corrected curves. (c) Cathodic chronoamperograms obtained by alternating two K^+^ concentrations, 50 and 1000 nM in 10 mM NaCl using the same ITO-POT-membrane electrode. The following sequence was followed: 1 → 10 → 1 → 10 → 1 → 10 μM KCl. (d) The corresponding cathodic coulometric corrected curves.

### Validated Analytical Application in Real Samples

The analytical applicability of the sensor was evaluated by measuring the K^+^ concentration in four different samples including urine, blood serum, canal water and a standard KCl solution. These samples were diluted with 10 mM NaCl to cover the linear range of response found in the different methods while ensuring the presence of an appropriate background electrolyte. Although this may not represent the optimal scenario for a decentralized application yet, the concept herein developed offers a promising and original approach with various advantages over potentiometric ISEs (e.g., logarithmic dependency of the ion activity, temperature influence, and membrane kinetics). Moreover, even with sample dilution, the consideration of different real matrices contributes to validating the applicability and potential of the coulometric readout, supporting its reliability for the further detection of other ions. The standard addition method was used for the A-IT-C protocol due to its operation in the nanomolar range, to account for possible errors caused by traces of K^+^ that may be present in the electrochemical cell. In contrast, the C-IT-C protocol, which operates in the micromolar range, is less affected by such residual K^+^ amounts. Therefore, an external calibration was employed for this protocol.

The K^+^-selective membrane herein used exhibits the same selectivity pattern as a conventional valinomycin-based potentiometric ISEs.^34^ This implies that ions such as Na^+^, H^+^, Ca^2+^, and Mg^2+^ will not interfere within the potential window for the K^+^ transfer. However, NH_4_^+^ may represent a potential interference in specific matrices such as urine, where its relative concentration to K^+^ is significant (K^+^/NH_4_^+^ ∼ 10) considering the reported logarithmic selectivity coefficient ( $\log K_{K^{+},NH_{4}^{+}}^{pot}=-1.7)$ . In the case of the A-IT-C protocol, any matrix effect is considered in the standard addition method, whereas it is necessary to understand the possibility for NH_4_^+^ interference in the C-IT-C protocol when comparing with an external calibration graph. First, we confirmed that that presence of NH_4_^+^ did not affect the transfer potential for K^+^ (0.99 V for K^+^, 0.87 V for NH_4_^+^ and 0.99 V for a 10/1 mixture of K^+^/NH_4_^+^, Figure S11). Then, we run the C-IT-C protocol to investigate any deviation in the K^+^ charge registered in the absence and presence of NH_4_^+^ (Figure S12). The results confirmed that the presence of NH_4_^+^ in the ratios expected in urine did not cause any significant change in the integrated charge, remaining this within the electrode’s percentage of variation (<7%).

Both protocols were performed with three independent ITO-POT-membrane electrodes to check the reproducibility of the analysis. As an example, the baseline-corrected LSV, chronoamperograms and the corresponding coulometric corrected curves for urine and serum using the anodic and cathodic protocols, are presented in Figure 8. The rest of the samples are shown in Figure S9 and Figure S10 in the Supporting Information.

**Figure 8:**
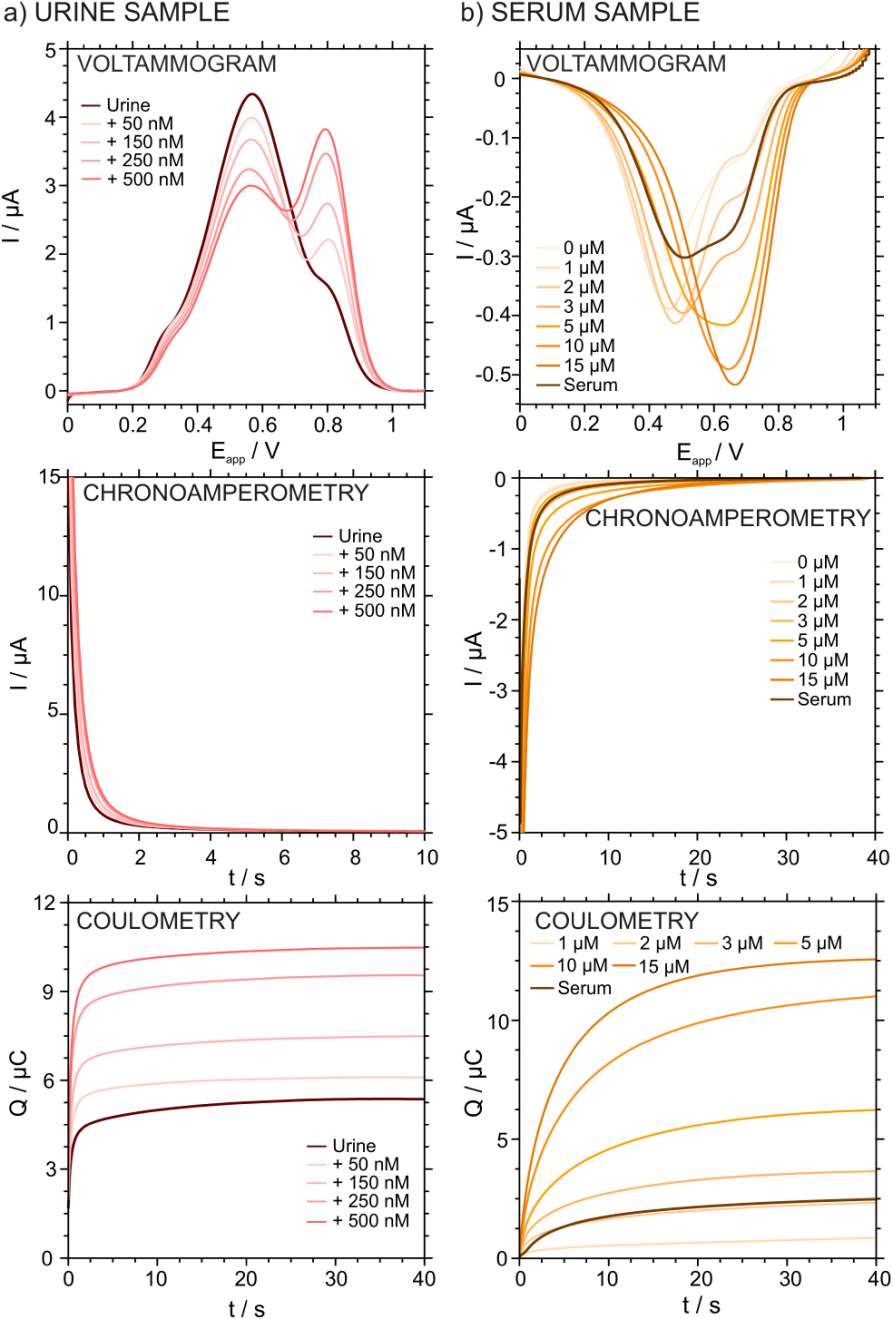
(a) Anodic LSVs, chronoamperograms and coulometric corrected curves for the urine sample. (b) Cathodic LSVs, chronoamperograms and coulometric corrected curves for the serum sample.

In the case of the urine analyzed with the A-IT-C protocol, the initial measurement corresponding to the diluted sample displayed two peaks. The first peak appeared in the range of 558–571 mV and is attributed to cations different from K^+^ present in the urine matrix. The second peak located between 793 and 806 mV, purely corresponded to K^+^. Upon standard additions of K^+^, the second peak increased in current, while the first peak decreased proportionally. In parallel, the current signal recorded at the K^+^ transfer potential (i.e., *E*_K_) was integrated to calculate the coulometric curves, which showed a gradual increase in charge with rising K^+^ concentrations.

For the serum sample analyzed with the C-IT-C protocol, an initial calibration was performed, with the sample measured at the end of the sequence. The LSV displayed two partially overlapping peaks: the first, appearing between 445 and 484 mV, is attributed mainly to Na^+^, while the second, at higher potentials, appearing in the range of 650–710 mV, corresponds to K^+^.

Table 1 presents the results for the K^+^ concentration quantification in each sample using all the possible measurements: (i) the peak current and charge from the anodic LSV, (ii) the peak current and charge from the cathodic LSV, (iii) the charge from the anodic chronoamperogram and (iv) the charge from the cathodic chronoamperogram. In addition, all the samples were analyzed by IC, with the correlations found between the results obtained with each technique and those provided by the IC shown in Figure S13 (Supporting Information). Moreover, Figure S14 depicts the Bland-Altman plots to evaluate the differences found between all the methods considered in pairs. The K^+^ amount in the standard solution was closer to the concentration provided by the supplier (100 mM) using all the methods (<6% of difference).

**Table 1:** Concentration of K^+^ Analyzed in Various Real Samples

	K^+^ concentration (mM), average ± SD (*n* = 3)						
	LSV A-IT-C		LSV C-IT-C				
Sample	*I*_peak_	*Q*_peak_	*I*_peak_	*Q*_peak_	Chrono A-IT-C	Chrono C-IT-C	C_IC_
Standard KCl	99.5 ± 8.4	100.0 ± 15.0	94.8 ± 5.8	96.5 ± 10.4	101.6 ± 9.2	98.8 ± 2.3	97.8
Urine	33.1 ± 5.9	29.1 ± 11.8	32.5 ± 4.8	39.6 ± 10.4	41.1 ± 3.1	36.0 ± 3.9	38.7
Serum	4.19 ± 1.25	2.22 ± 1.19	3.13 ± 0.41	3.09 ± 1.55	5.13 ± 0.43	2.51 ± 0.15	2.49
Canal water	0.30 ± 0.03	0.26 ± 0.19	0.43 ± 0.02	0.45 ± 0.03	0.50 ± 0.02	0.45 ± 0.03	0.46

For the biological samples, the K^+^ levels were within the expected physiological ranges (20–125 mM in human urine^35^ and 2.4–4.8 mM in equine serum^36^). However, the values obtained using chrono A-IT-C and LSV A-IT-C in terms of *I*_peak_ for serum appear to be significantly higher than those obtained with the other methods. Notably, the LSV-based methods exhibited higher variability, with RSD for charge-based analyses (*Q*_peak_) reaching approximately 50% for both anodic and cathodic approaches, whereas this magnitude was below 10% for the chronoamperometry. This improved consistency may be attributed to the simplest data processing associated with chronoamperometry. Once more, the anodic protocol appeared more susceptible to trace interference than the cathodic one, as previously observed.

The results obtained analyzing the *I*_peak_ from both LSV protocols showed mean values closely aligned with IC results, with mean bias below 3 mM. Despite a strong Pearson correlation coefficient (value of 0.9972), Bland-Altman analysis revealed notable deviations, particularly for the canal water and serum samples. Similarly, the *Q*_peak_ readout from LSV C-IT-C and A-IT-C showed biases of −0.5 mM and −2 mM respectively with a Pearson coefficient of 0.9999. Overall, LSV measurements demonstrated a higher dispersion than the chronoamperometric approaches, especially under anodic interrogation.

Chronoamperometric methods exhibited the most consistent and accurate performance in both the anodic and cathodic interrogation. As previously mentioned, chrono A-IT-C displayed significant overestimation for the serum sample compared to the IC values (ca. + 75% of difference). However, results for the other samples exhibited a good agreement with the IC, as indicated by a mean bias of +2.1 mM and Pearson coefficient of 0.9998. Chrono C-IT-C, demonstrated the best overall concordance with IC results, showing minimal mean difference (−0.4 mM) and excellent correlation (Pearson coefficient of 0.9994) across all the tested samples. In general, both chronoamperometric routines exhibited excellent agreement with IC, presenting mean relative differences below 7% for all the analyzed samples (excluding the serum measured with A-IT-C).

In summary, the chronoamperometry routines provided more consistent and reliable quantification compared to LSV, with proven suitability for determining K^+^ in the various real samples. C-IT-C showed the highest agreement with IC, likely due to its simpler protocol that avoids the need for selective Na^+^ (interferent cation) expulsion prior to K^+^ uptake into the membrane. These findings confirm the analytical capability of the developed sensor for detecting K^+^ in complex matrices, covering a wide concentration range from nanomolar to micromolar levels. From a biological perspective, the tunable nature of the proposed coulometric methodology could be particularly advantageous for ions which concentration varies drastically across different biological compartments. A representative example is the calcium analysis in living cells: while Ca^2+^ is present at millimolar concentrations in serum, its intracellular concentration (e.g., in erythrocytes) lies in the nanomolar range. Extending the present concept to such systems represents an exciting and highly relevant direction for future research.

## Conclusions

We have explored two charge-based protocols for detecting K^+^ in tunable concentration ranges spanning from the nanomolar to the micromolar level. The same sensing platforms can be adapted to different concentration windows by selecting anodic or cathodic interrogation, while preserving accuracy and reproducibility. On one hand, the anodic approach (A-IT-C) enables the detection of K^+^ in the nanomolar range from 200 to 1000 nM, while on the other hand, the cathodic protocol (C-IT-C) covers the micromolar range from 3 to 20 μM. Both interrogation approaches presented good repeatability and reversibility throughout measurements. The C-IT-C interrogation provided a faster and more efficient response, since K^+^ can be directly accumulated into the membrane. In contrast, the A-IT-C protocol required the prior expulsion of Na^+^ (or any interfering cation present in the sample). Also, it has been demonstrated that the charge associated with the K^+^ transfer at the membrane-sample interface significantly depends on the amount of K^+^ in the bulk solution, while the total charge in the system remains constant. Finally, the analytical applicability of the sensor was validated with real samples of different nature including urine, serum, water sample and a standard solution. It was found that both chronoamperometric protocols, A-IT-C and C-IT-C, exhibited results in good agreement with those obtained by using IC though and statistical analysis considering Bland–Altman and correlation plots. Remarkably, the C-IT-C protocol is shorter and operationally simpler, making it potentially suitable for high-frequency measurements. Future work in our research group is expected in several directions, including the integration of thin-layer samples as well as numerical simulations to study how to adjust the linear range of response to guide the analysis of any real sample using the same electrode.

## Supplementary Material



## Notes

Experimental setup illustration; data treatment for charge calculation; raw data; statistical analysis; repeatability and reversibility data; selectivity study towrds NH_4_^+^ (PDF)

## References

[1] Kabagambe B., Izadyar A., Amemiya S. (2012). Stripping Voltammetry of Nanomolar
Potassium and Ammonium Ions Using a Valinomycin-Doped Double-Polymer
Electrode. Anal. Chem..

[2] Cuartero M., Crespo G. A., Bakker E. (2015). Thin Layer
Samples Controlled by
Dynamic Electrochemistry. Chimia.

[3] Yuan D., Cuartero M., Crespo G. A., Bakker E. (2017). Voltammetric Thin-Layer
Ionophore-Based Films: Part 2. Semi-Empirical Treatment. Anal. Chem..

[4] Cuartero M., Crespo G. A. (2018). All-Solid-State Potentiometric Sensors:
A New Wave
for in Situ Aquatic Research. Curr. Opin. Electrochem..

[5] Zdrachek E., Bakker E. (2019). Potentiometric Sensing. Anal.
Chem..

[6] Crespo G. A., Cuartero M., Bakker E. (2015). Thin Layer Ionophore-Based
Membrane
for Multianalyte Ion Activity Detection. Anal.
Chem..

[7] Cuartero M., Crespo G. A., Bakker E. (2016). Ionophore-Based Voltammetric
Ion
Activity Sensing with Thin Layer Membranes. Anal. Chem..

[8] Kim Y., Amemiya S. (2008). Stripping Analysis of Nanomolar Perchlorate in Drinking
Water with a Voltammetric Ion-Selective Electrode Based on Thin-Layer
Liquid Membrane. Anal. Chem..

[9] Kim Y., Rodgers P. J., Ishimatsu R., Amemiya S. (2009). Subnanomolar Ion Detection
by Stripping Voltammetry with Solid-Supported Thin Polymeric Membrane. Anal. Chem..

[10] Izadyar A., Al-Amoody F., Arachchige D. R. (2016). Ion Transfer Stripping Voltammetry
to Detect Nanomolar Concentrations of Cr (VI) in Drinking Water. J. Electroanal. Chem..

[11] Crespo G. A., Ghahraman Afshar M., Dorokhin D., Bakker E. (2014). Thin Layer Coulometry
Based on Ion-Exchanger Membranes for Heparin Detection in Undiluted
Human Blood. Anal. Chem..

[12] Liu Y., Crespo G. A., Cuartero M. (2022). Spectroelectrochemistry
with Ultrathin
Ion-Selective Membranes: Three Distinct Ranges for Analytical Sensing. Anal. Chem..

[13] Yuan D., Cuartero M., Crespo G. A., Bakker E. (2017). Voltammetric Thin-Layer
Ionophore-Based Films: Part 1. Experimental Evidence and Numerical
Simulations. Anal. Chem..

[14] Xu K., Cuartero M., Crespo G. A. (2019). Lowering
the Limit of Detection of
Ion-Selective Membranes Backside Contacted with a Film of Poly­(3-Octylthiophene). Sens. Actuators, B Chem..

[15] Shi C., Anson F. C. (1998). A Simple Method for Examining the Electrochemistry
of Metalloporphyrins and Other Hydrophobic Reactants in Thin Layers
of Organic Solvents Interposed between Graphite Electrodes and Aqueous
Solutions. Anal. Chem..

[16] Guo J., Amemiya S. (2006). Voltammetric Heparin-Selective
Electrode Based on Thin
Liquid Membrane with Conducting Polymer-Modified Solid Support. Anal. Chem..

[17] Si P., Bakker E. (2009). Thin Layer Electrochemical
Extraction of Non-Redoxactive
Cations with an Anion-Exchanging Conducting Polymer Overlaid with
a Selective Membrane. Chem. Commun..

[18] Liu Y., Crespo G. A., Cuartero M. (2024). Voltammetric
Ion-Selective Electrodes
in Thin-Layer Samples: Absolute Detection of Ions Using Ultrathin
Membranes. Anal. Chem..

[19] Liu Y., Crespo G. A., Cuartero M. (2025). Approaching
to Calibration-Free Ion
Detection Based on Thin Layer Coulometry with Ultrathin Ion-Selective
Membranes. ACS Meas. Sci. Au.

[20] Han T., Mousavi Z., Mattinen U., Bobacka J. (2020). Coulometric Response
Characteristics of Solid Contact Ion-Selective Electrodes for Divalent
Cations. J. Solid State Electrochem..

[21] Han T., Mattinen U., Mousavi Z., Bobacka J. (2021). Coulometric Response
of Solid-Contact Anion-Sensitive Electrodes. Electrochim. Acta.

[22] Han T., Mattinen U., Bobacka J. (2019). Improving the Sensitivity of Solid-Contact
Ion-Selective Electrodes by Using Coulometric Signal Transduction. ACS Sensors.

[23] Vanamo U., Hupa E., Yrjänä V., Bobacka J. (2016). New Signal
Readout Principle for Solid-Contact Ion-Selective Electrodes. Anal. Chem..

[24] Jarolímová Z., Han T., Mattinen U., Bobacka J., Bakker E. (2018). Capacitive Model for
Coulometric Readout of Ion-Selective Electrodes. Anal. Chem..

[25] Ding J., Qin W. (2009). Current-Driven Ion Fluxes of Polymeric
Membrane Ion-Selective Electrode
for Potentiometric Biosensing. J. Am. Chem.
Soc..

[26] Liu S., Ding J., Qin W. (2018). Current Pulse Based Ion-Selective
Electrodes for Chronopotentiometric Determination of Calcium in Seawater. Anal. Chim. Acta.

[27] Li J., Zhang W., Qin W. (2023). Trace-Level Chronopotentiometric
Detection in the Presence of a High Electrolyte Background Using Thin-Layer
Ion-Selective Polymeric Membranes. Chem. Commun..

[28] Nussbaum R., Jeanneret S., Bakker E. (2024). Increasing the Sensitivity of PH
Glass Electrodes with Constant Potential Coulometry at Zero Current. Anal. Chem..

[29] Wang H., Yuan B., Yin T., Qin W. (2020). Alternative Coulometric
Signal Readout Based on a Solid-Contact Ion-Selective Electrode for
Detection of Nitrate. Anal. Chim. Acta.

[30] Liu Y., Wiorek A., Crespo G. A., Cuartero M. (2020). Spectroelectrochemical
Evidence of Interconnected Charge and Ion Transfer in Ultrathin Membranes
Modulated by a Redox Conducting Polymer. Anal.
Chem..

[31] Cuartero M., Crespo G. A., Bakker E. (2016). Polyurethane Ionophore-Based
Thin
Layer Membranes for Voltammetric Ion Activity Sensing. Anal. Chem..

[32] Long R., Bakker E. (2004). Optical determination
of ionophore diffusion coefficients
in plasticized poly (vinyl chloride) sensing films. Anal. Chim. Acta.

[33] Cuartero M., Acres R. G., De Marco R., Bakker E., Crespo G. A. (2016). Electrochemical
Ion Transfer with Thin Films of Poly­(3-octylthiophene). Anal. Chem..

[34] Liu Y., Crespo G. A., Cuartero M. (2021). Semi-empirical
treatment of Ionophore-assisted
ion-transfers in Ultrathin Membranes Coupled to a redox Conducting
Polymer. Electrochim. Acta.

[35] UCSF Health. Potassium Urine Test. https://www.ucsfhealth.org/medical-tests/potassium-urine-test 2024.

[36] Cornell University College of Veterinary Medicine. Chemistry. https://www.vet.cornell.edu/animal-health-diagnostic-center/laboratories/clinical-pathology/reference-intervals/chemistry 2024.

