# Supplementary material for: Tunable Ion-Sensing Using Coulometric-Based Protocols with Permselective Nanomembranes
Source: Anal Chem. 2026 Feb 2;98(6):4972–82. doi: 10.1021/acs.analchem.5c07283 (PMC12921655; doi:10.1021/acs.analchem.5c07283)
Supplement: Supplementary file 1 [file ac5c07283_si_001.pdf]

Supporting Information for:

Tunable Ion-Sensing using Coulometric-Based Protocols with Permselective Nanomembranes

Nuria Martínez-Lorca[1], Yujie Liu[2], Gregorio Laucirica[1], Gastón A. Crespo[1,2], María Cuartero[1,2,*]

[1]UCAM-SENS, Universidad Católica San Antonio de Murcia, UCAM HiTech, Avda. Andrés Hernández Ros 1, 30107, Murcia, Spain.

[2]Department of Chemistry, KTH Royal Institute of Technology, Teknikringen 30, SE-114 28, Stockholm, Sweden.

*Corresponding author: mariacb@kth.se

KEYWORDS: Ion-selective electrode, poly(3-octylthiophene), nanomembrane, coulometry, thin-layer electrochemistry.

Table of Contents

Figures........ 3

Figure S1. Experimental setup 3

Protocol for charge calculation. 4

Figure S2. Data treatment for charge calculation from LSV 4

Figure S3. Data treatment for charge calculation from chronoamperometry 5

Figure S4. Original and uncorrected LSV anodic data at increasing KCl concentrations 6

Figure S5. Anodic chronoamperograms when $E_{baseline}$ is applied at increasing KCl concentrations 6

Figure S6. Plot of the chrono-charge versus LSV-charge at increasing KCl concentrations 7

Figure S7. Original and uncorrected LSV cathodic data at increasing KCl concentrations 8

Figure S8. Cathodic chronoamperograms when $E_{baseline}$ is applied at increasing KCl concentrations 8

Figure S9. Serum, canal water and standard solution evaluated with anodic protocol using standard additions method 9

Figure S10. Urine, canal water and standard solution evaluated with cathodic protocol using an external calibration 10

Figure S11. Cyclic voltammograms in 10 mM NH_4Cl, 10 mM KCl, and a mixed solution containing 1 mM NH_4Cl and 10 mM KCl 11

Figure S12. Coulometric responses for a 3 μM KCl solution and for the same solution after adding 0.4 μM NH_4Cl 11

Figure S13. Correlation between concentrations determined by different techniques and ion chromatography 13

Figure S14. Bland-Altman plots of the difference of concentrations 14

Tables........ 15

Table S1. Repeatability for two different concentrations using both protocols 15

Table S2. Reversibility for two concentrations using both protocols following the sequence lowest → highest concentration. 15

Figures

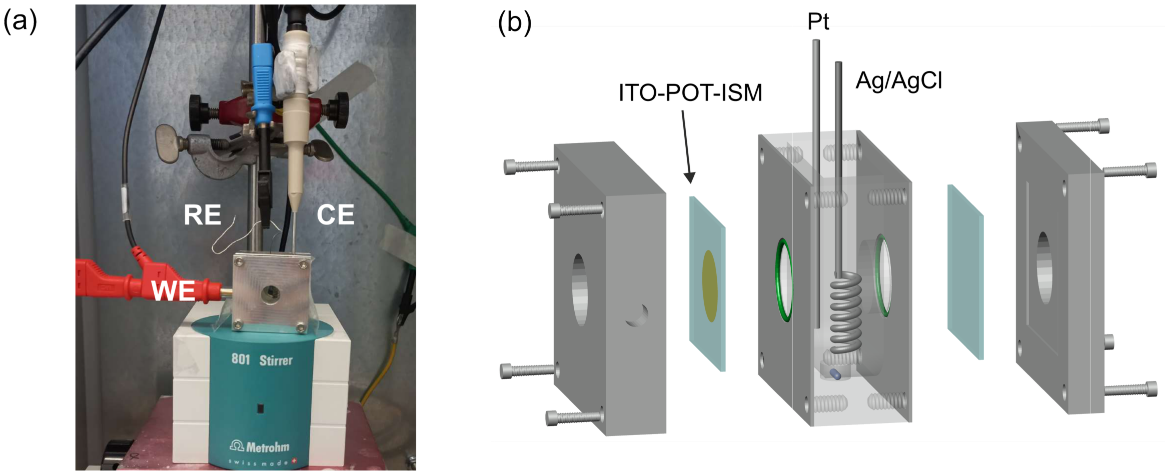

Figure S1. Experimental setup **(a)** Photograph of the electrochemical setup. WE: working electrode, RE: reference electrode, CE: counter electrode. **(b)** Schematic representation of the electrochemical cell.

Protocol for charge calculation.

For the LSV data, which involved two overlapping peaks, a baseline correction was first applied to the voltammograms, followed by peak deconvolution using Gaussian fitting. The charge associated with each peak was then calculated by integrating the area under the fitted curve and dividing it by the scan rate (see Figure S2 as an example). Additionally, to ensure accurate quantification, any residual charge contribution from background K^+ levels likely due to minor contamination in the initial solution was systematically subtracted from all calibration curves.

For the chronoamperometric data, a simple baseline correction was applied followed by direct integration of the current signal with the later subtraction of the charge from non-faradaic processes (i.e. charge corresponding to the application of $E_{baseline}$) (see Figure S3 as an example). Additionally, the residual signal associated with the background level of K^+ was systematically removed from all curves to ensure that each measurement started from a baseline corresponding to a fully cleaned signal.

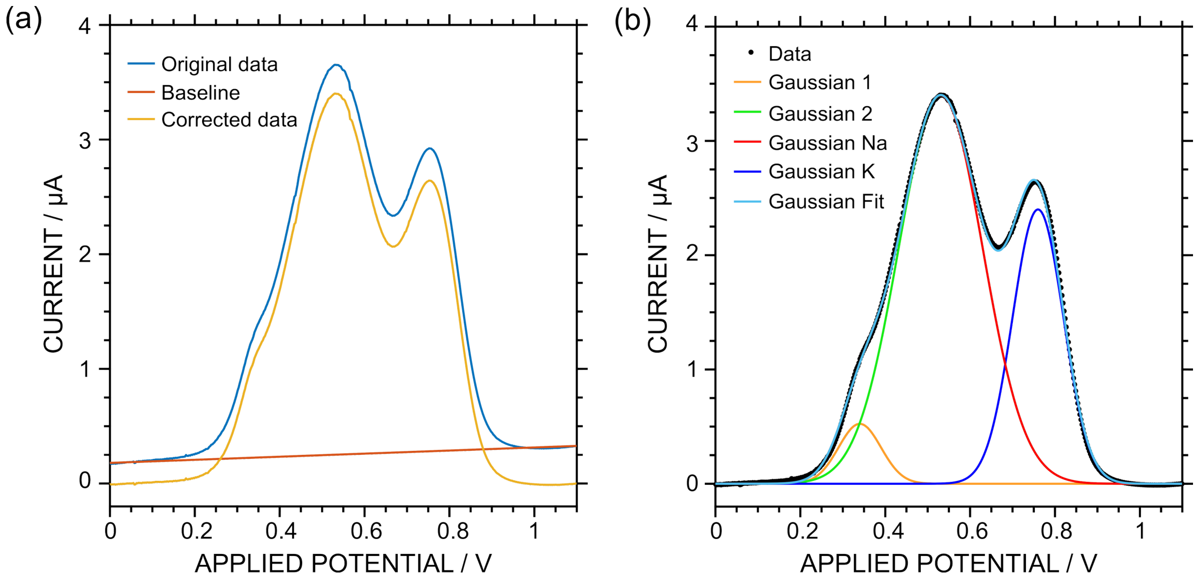

Figure S2. Data treatment for charge calculation from LSV **(a)** Baseline correction for LSV. **(b)** Gaussian fit for the calculation of charge for Na^+ and K^+ transfer peaks.

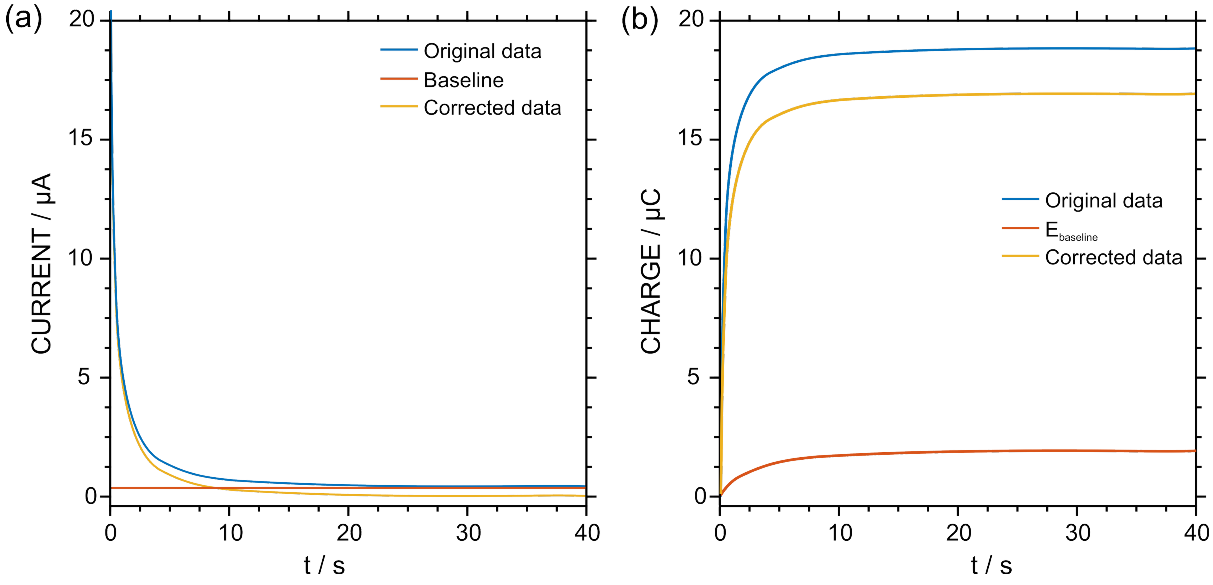

Figure S3. Data treatment for charge calculation from chronoamperometry **(a)** Baseline correction for chronoamperometry. **(b)** Integration of the current signal and subtraction of the charge associated with non-faradaic processes.

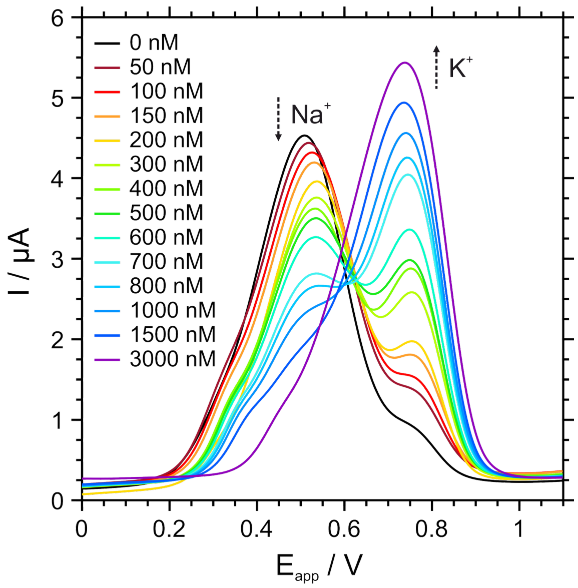

Figure S4. Original and uncorrected LSV anodic data at increasing KCl concentrations in 10 mM NaCl background. Electrochemical protocol: E_{app} = −0.2 V during 450 s, stirring speed of 100 rpm, LSV from −0.2 to 1.1 V, scan rate of 50 mV s^{-1}.

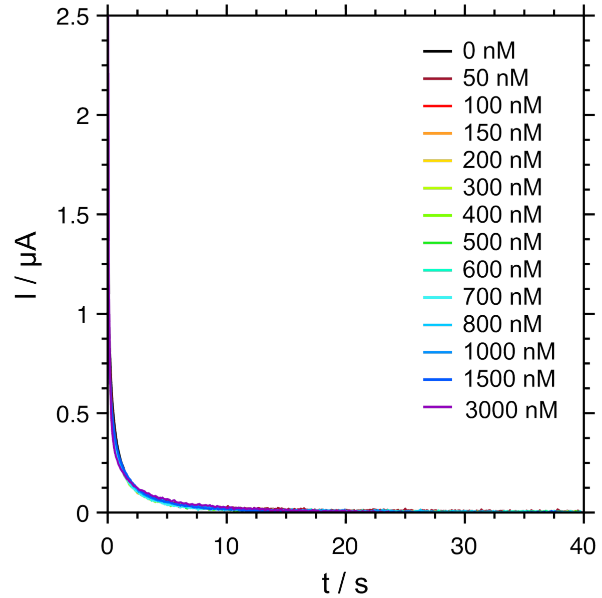

Figure S5. Anodic chronoamperograms when $E_{baseline}$ is applied at increasing KCl concentrations in 10 mM NaCl solution at 0.10 V.

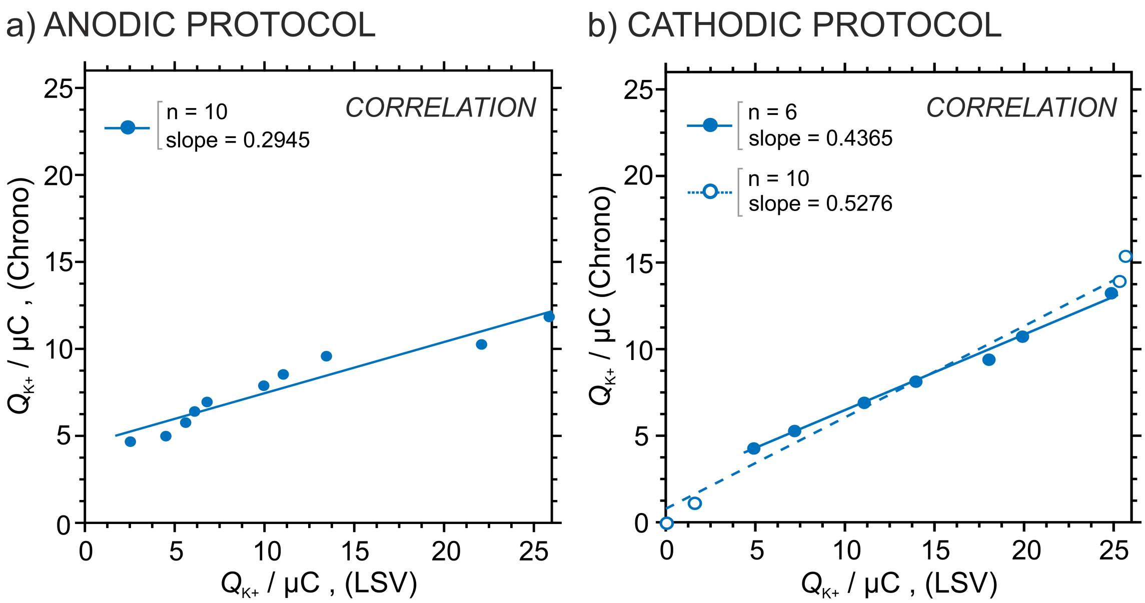

Figure S6. Plot of the chrono-charge versus LSV-charge at increasing KCl concentrations in 10 mM NaCl solution for **(a)** Anodic and **(b)** Cathodic protocol.

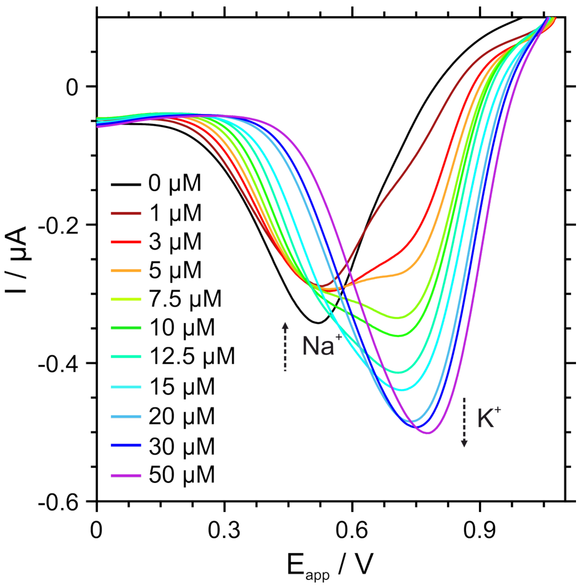

Figure S7. Original and uncorrected LSV cathodic data at increasing KCl concentrations in 10 mM NaCl background. Electrochemical protocol: E_{app} = 1 V during 150 s, stirring speed of 100 rpm, LSV from 1.1 to −0.1 V, scan rate of 5 mV s^{-1}.

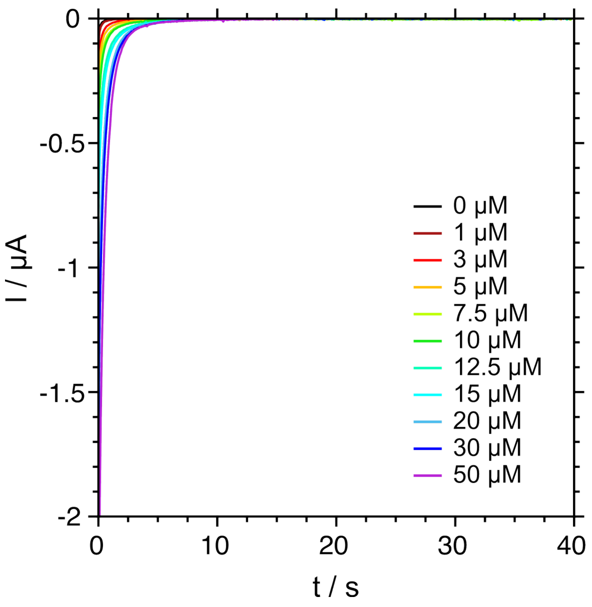

Figure S8. Cathodic chronoamperograms when $E_{baseline}$ is applied at increasing KCl concentrations in 10 mM NaCl solution at 0.95 V.

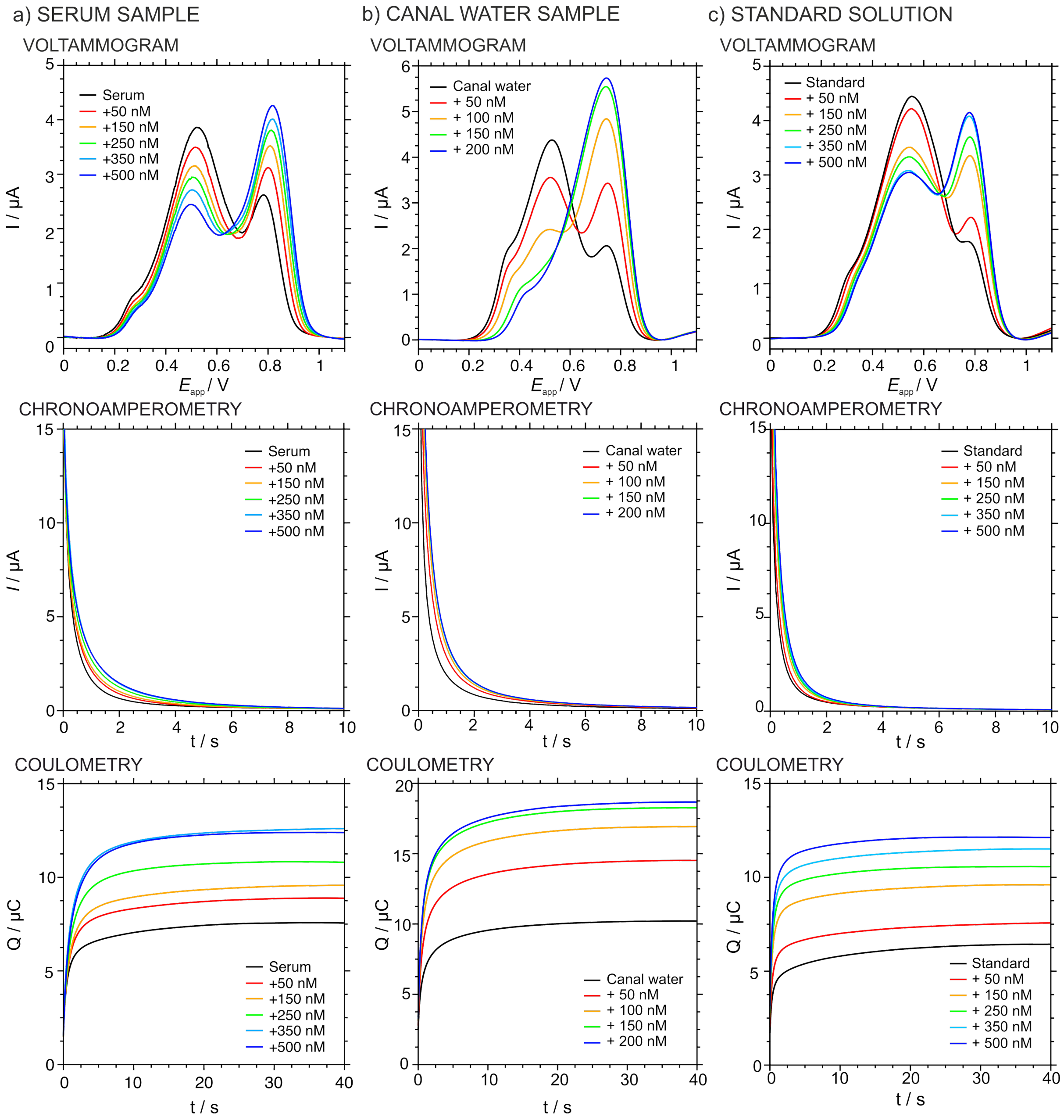

Figure S9. Serum, canal water and standard solution evaluated with anodic protocol using standard additions method in 10 mM NaCl background. **(a)** LSV, chronoamperograms and coulometric corrected signals for serum sample. **(b)** LSV, chronoamperograms and coulometric corrected signals for canal water. **(c)** LSV, chronoamperograms and coulometric corrected signals for standard KCl. Electrochemical protocol: E_{app} = –0.2 V during 450 s, stirring speed of 100 rpm, LSV from –0.2 to 1.1 V, scan rate of 50 mV s^{-1}, E_{app} = –0.2 V during 450 s, stirring speed of 100 rpm, $E_{baseline}$ = 0.10 V during 40 s, E_{Na} = 0.50 V during 40 s, E_K = 0.80 V during 40 s.

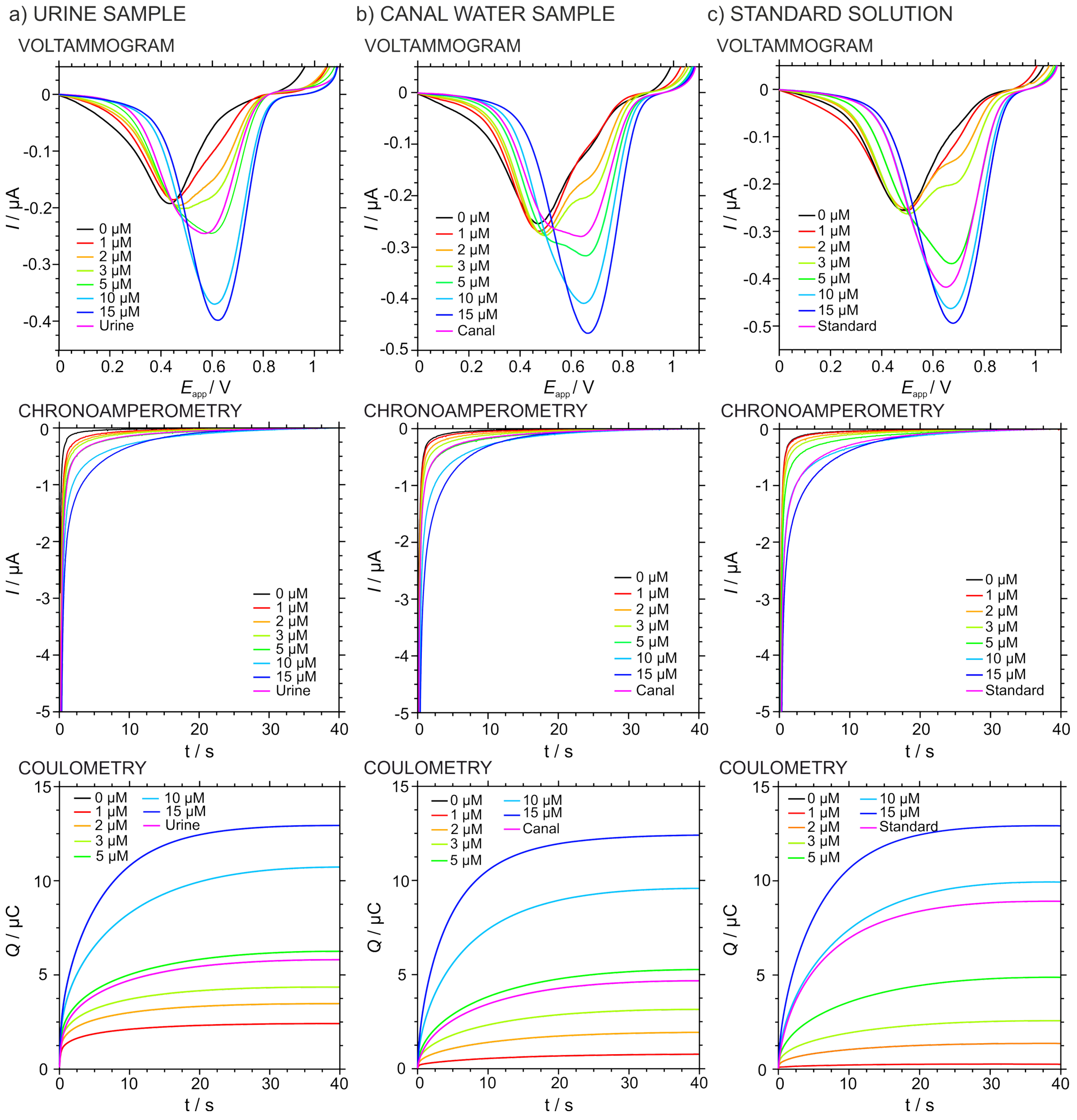

Figure S10. Urine, canal water and standard solution evaluated with cathodic protocol using an external calibration in 10 mM NaCl background (**a**) LSV, chronoamperograms and coulometric corrected signals for urine sample. (**b**) LSV, chronoamperograms and coulometric corrected signals for canal water. (**c**) LSV, chronoamperograms and coulometric corrected signals for standard KCl. Electrochemical protocol: E_{app} = 1 V during 150 s, stirring speed of 100 rpm, LSV from 1.1 to –0.1 V, scan rate of 5 mV s^{-1}, E_{app} = 1 V during 150 s, stirring speed of 100 rpm, $E_{baseline}$ = 0.95 V during 40 s, E_K = 0.65 V during 40 s.

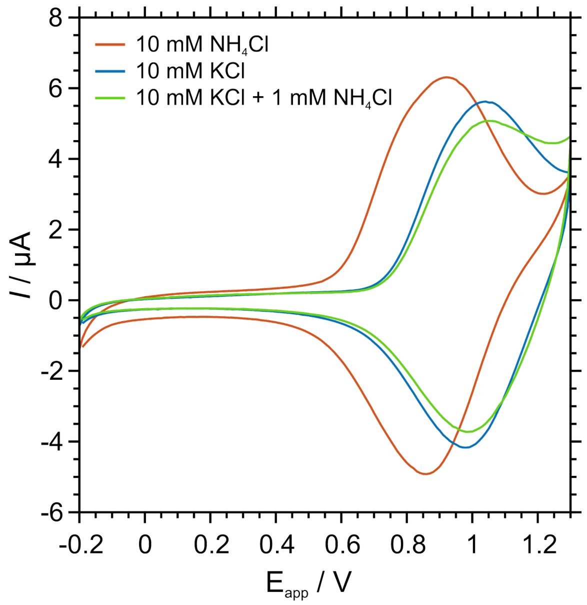

Figure S11. Cyclic voltammograms in 10 mM NH_4Cl (red line), 10 mM KCl (blue line), and a mixed solution containing 1 mM NH_4Cl and 10 mM KCl (green line). Scan rate: 100 mV s^{-1}.

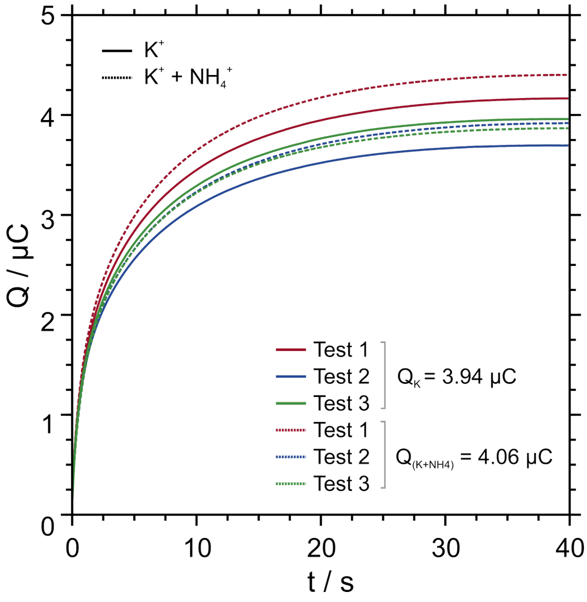

Figure S12. Coulometric responses for a 3 µM KCl solution (solid lines) and for the same solution after adding 0.4 µM NH_4Cl (dashed lines). Sequential measurements were performed with the same electrode to assess the possible influence of NH_4^+ on the K^+-transfer charge at the highest ratio expected in the urine sample (1:10) with respect to the natural variation of the sensor response upon consecutive measurements. C-IT-C protocol: Application of $E_K = 0.65$ V for 40 s.

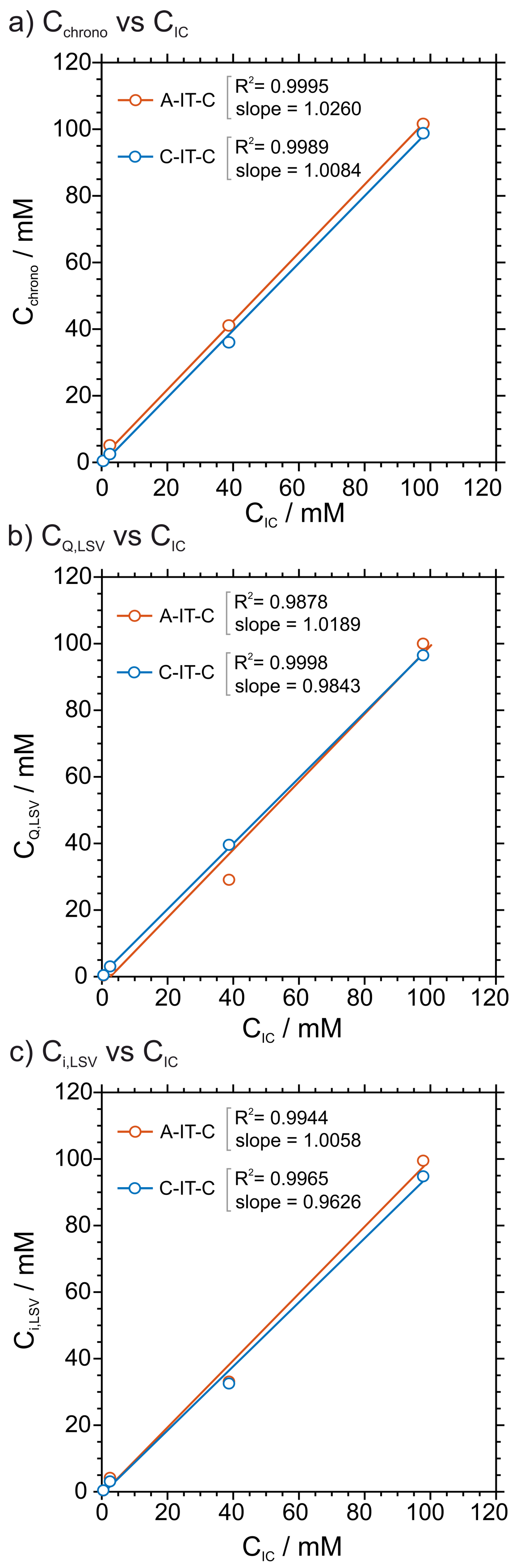
a) C_chrono vs C_IC
A-IT-C
R²= 0.9995
slope = 1.0260
C-IT-C
R²= 0.9989
slope = 1.0084
C_chrono / mM
C_IC / mM
b) C_Q,LSV vs C_IC
A-IT-C
R²= 0.9878
slope = 1.0189
C-IT-C
R²= 0.9998
slope = 0.9843
C_Q,LSV / mM
C_IC / mM
c) C_i,LSV vs C_IC
A-IT-C
R²= 0.9944
slope = 1.0058
C-IT-C
R²= 0.9965
slope = 0.9626
C_i,LSV / mM
C_IC / mM

Figure S13. Correlation between concentrations determined by different techniques and ion chromatography. (**a**) Qchrono, (**b**) Qpeak from LSV, and (**c**) Ipeak from LSV.

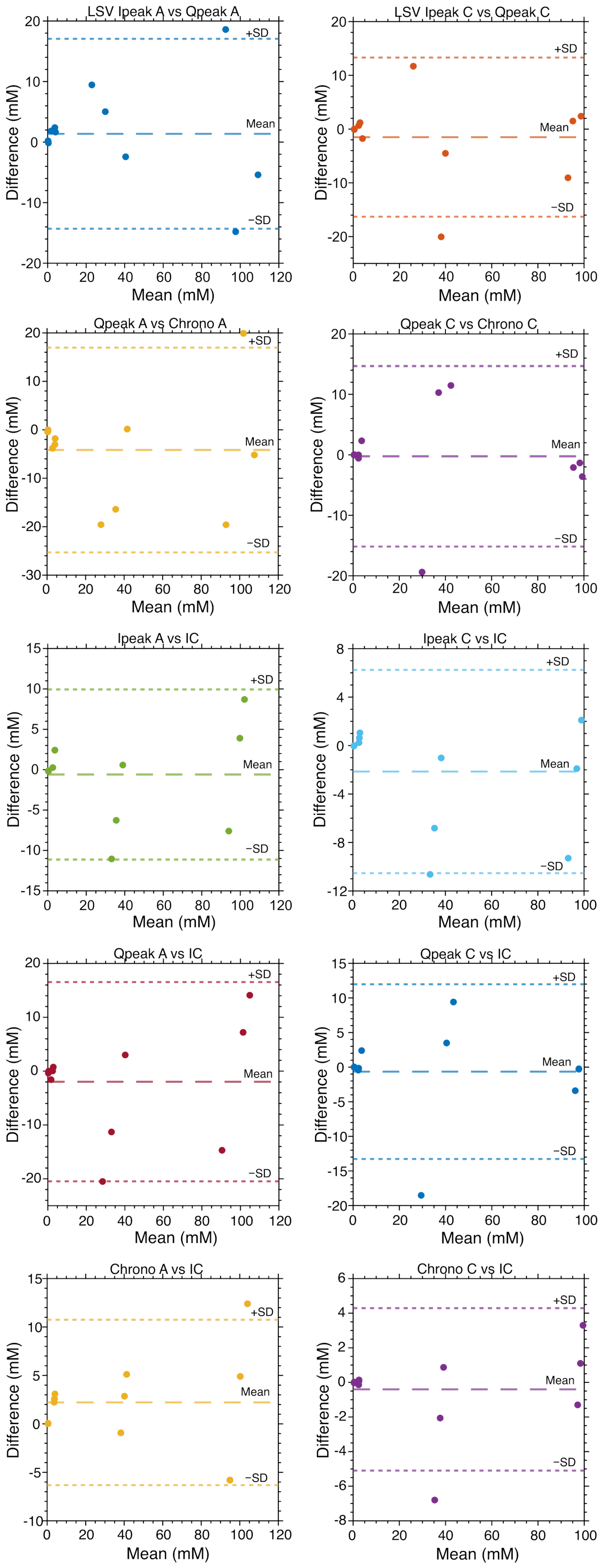

Figure S14. Bland-Altman plots of the difference of concentrations obtained between all the methods.

Tables

Table S1. Repeatability for two different concentrations using both protocols.

Repeatability	ANODIC CHARGE (μC)		CATHODIC CHARGE (μC)	
	50 nM	1000 nM	1 μM	12.5 μM
Measure #1	15.869	23.911	3.122	11.412
Measure #2	15.996	23.907	3.142	11.272
Measure #3	16.269	23.916	3.103	11.265
Average ± SD	16.04 ± 0.20	23.91 ± 0.00	3.12 ± 0.02	11.32 ± 0.08
RSD	1.27	0.02	0.63	0.73

Table S2. Reversibility for two concentrations using both protocols following the sequence lowest → highest concentration.

Reversibility	ANODIC CHARGE (μC)		CATHODIC CHARGE (μC)	
	50 nM	1000 nM	1 μM	10 μM
Measure 1	16.918	21.242	2.692	6.732
Measure 2	16.941	18.703	2.208	6.493
Measure 3	14.621	18.864	2.097	6.086
Average ± SD	16.16 ± 1.33	19.60 ± 1.42	2.33 ± 0.32	6.44 ± 0.33
RSD	8.25	7.25	13.55	5.07
